# Disease burden from tobacco consumption in Peru and the projected effect of strengthening control measures: a modeling study

**DOI:** 10.17843/rpmesp.2025.422.14338

**Published:** 2025-06-23

**Authors:** Ariel Bardach, Andrea Alcaraz, Jhonatan R. Mejia, Natalia Espinola, Elena Lazo, Federico Cairoli, Alfredo Palacios, Lucas Perelli, Federico Augustovski, Cesar Loza-Munarriz, Agustin Casarini, Andrés Pichon-Riviere

**Affiliations:** 1 Institute for Clinical and Health Effectiveness (IECS), Buenos Aires, Argentina Institute for Clinical and Health Effectiveness (IECS) Buenos Aires Argentina; 2 Center for Research in Epidemiology and Public Health (CIESP), Institute for Clinical and Health Effectiveness (IECS-CONICET), Buenos Aires, Argentina Center for Research in Epidemiology and Public Health (CIESP) Institute for Clinical and Health Effectiveness (IECS-CONICET) Buenos Aires Argentina; 3 Clinical Epidemiology Unit, Alberto Hurtado School of Medicine, Universidad Peruana Cayetano Heredia. Lima, Peru. Universidad Peruana Cayetano Heredia Clinical Epidemiology Unit Alberto Hurtado School of Medicine Universidad Peruana Cayetano Heredia Lima Peru

**Keywords:** Tobacco use, Tobacco Control, Cost of illness, Perú

## Abstract

**Objectives.:**

To estimate the economic burden and disease burden associated with tobacco use in Peru and the projected effect of strengthening specific tobacco control measures.

**Materials and methods.:**

We used a Markov microsimulation model to assess smoking-attributable mortality, disease events, economic costs, and projected benefits over ten years, considering the implementation of measures such as plain packaging, full enforcement of smoke-free laws, a complete ban on tobacco advertising, promotion, and sponsorship, and increased cigarette taxes.

**Results.:**

Each year in Peru, approximately 22,350 deaths and 126,000 disease events are attributable to tobacco use, accounting for 19% of all deaths from heart disease, 18% of deaths from stroke, and 515,768 years of life lost. In addition, approximately 1.28% of gross domestic product is lost annually due to smoking. Over ten years, the implementation of plain packaging could prevent 6,218 deaths, 31,700 events, and save 576 million USD. Full compliance with smoke-free places would prevent 4,982 deaths, 25,400 events, and save 461 million USD. Banning advertising, promotion, and sponsorship could prevent 8,767 deaths, 44,700 events, and save 812 million USD. Increasing cigarette prices by 50% could prevent 20,400 deaths, 658,400 healthy life years lost, and an economic benefit of more than 3.3 billion USD.

**Conclusions.:**

The economic and disease burden of tobacco use in Peru is significant. Greater efforts to control tobacco would significantly reduce this burden.

## INTRODUCTION

Tobacco use is responsible for more than 8 million deaths worldwide, making it the leading cause of disability-adjusted life years (DALYs) in men and the seventh leading cause in women in 2019 ^1^. Significant progress has been made in tobacco control worldwide since the implementation of the World Health Organization (WHO) Framework Convention on Tobacco Control (FCTC) and the associated MPOWER measures ^2^. However, 20.9% of the global population aged 15 years or older still uses tobacco ^3^, making tobacco use one of the leading preventable risk factors for morbidity and mortality worldwide. In addition, the economic burden associated with tobacco use is considerably high, accounting for 5.7% of global health expenditure ^4^.

Among WHO regions, the Americas have the second lowest prevalence in the world (16.3%) of age-adjusted tobacco use ^5^. However, smoking is estimated to cause around 14% of all deaths in the region ^6^. In the context of Latin America, the economic impact of tobacco use is significant, at USD 26.9 billion ^7^, and goes beyond direct healthcare costs to include social costs (labor productivity and informal care) ^7^. Specifically, Peru has one of the lowest adult smoking prevalences, with an estimated age-adjusted rate of 7.1% (11.6% in men and 2.6% in women) of Peruvian smokers ^3^. In addition, tobacco use among young people is relatively low, with an estimated age-adjusted prevalence of 7.2% of young smokers ^8^. Despite the low prevalence of smoking in Peru, a previous study showed that 31% of deaths from smoking-related diseases were attributed to tobacco in 2015 ^9^, with 22,374 deaths and an estimated economic burden of USD 2.651 billion in 2020 ^10^.

The pillars of tobacco control in Peru are Laws 28705 (2006) ^11^^)^ and 29517 (2010) ^12^, passed after the ratification in 2004 of the WHO FCTC ^2^, which is legally considered a human rights treaty in the country. These laws established measures related to tobacco control based on protection against exposure to tobacco smoke, a ban on smoking in enclosed spaces, and regulations on advertising and sponsorship. In this way, Peru has implemented a smoking ban in several enclosed places and certain private areas, as well as in some outdoor public spaces, achieving substantial compliance with this regulation (obtaining a score of 8 out of 10 according to the WHO) ^13^. However, there is no explicit ban on all outdoor public areas. With regard to packaging and labeling, comprehensive warnings (50% of the front and back) with all the appropriate features (e.g., rotation, inclusion of images) have been added to cigarette packs. In addition, although taxes on cigarettes have increased, they currently represent only about 73.3% of the retail price ^13^, below the WHO recommendation of 75%, and cigarettes are no less affordable than a decade ago. On the other hand, the least implemented measure is the ban on advertising, promotion, and sponsorship, as there is no comprehensive ban and forms of direct and indirect advertising are still permitted (e.g., in magazines for people over 18 years of age). The implementation of these measures has contributed to reducing the prevalence of smoking, as evidenced by a reduction in active smoking of more than 6% in males and 12% in female adolescents from 2007 to 2019 ^14^. However, one study reported a small reduction in prematurity after the implementation of tobacco control laws, suggesting that these measures should be strengthened ^15^.

Therefore, despite progress in tobacco control in Peru, the disease and economic burden of smoking in the country may still be high, as evidenced by our previous model with data up to 2015 ^9^. In addition, there is still room to improve and strengthen tobacco control measures (e.g., plain packaging, tax increases), which could reduce national costs and the disease burden. In this regard, our previous model only assessed the impact of price increases through taxes, without considering other tobacco control measures ^9^. For these reasons, this study aimed to estimate the economic and disease burden attributable to tobacco use in Peru by extending the previous model to 2020, as well as the potential reductions in these burdens that could be achieved with the implementation and strengthening of key tobacco control measures, such as plain packaging with more than 80% of the surface area covered by health warnings, full compliance with smoke-free laws, and a complete ban on tobacco advertising, promotion, and sponsorship (TAPS).

**KEY MESSAGES**

Motivation for the study. Despite progress in tobacco control, the economic and disease burden in Peru remains high. Strengthening smoke-free regulations, implementing plain packaging, banning tobacco promotion and sponsorship, and increasing taxes could reduce it.

Main findings. Tobacco use causes 22,350 deaths and 126,000 disease events annually in Peru, resulting in a loss of 1.28% of GDP. Strengthening tobacco control policies would prevent thousands of deaths and save billions in costs.

Implications. Stricter tobacco control policies can reduce the health and economic costs associated with smoking for the advancement of public health and economic sustainability in Peru.

## MATERIALS AND METHODS

### Economic model

This is a modeling study using a first-order Markov disease and economic model developed at the Institute for Clinical and Health Effectiveness (IECS, Buenos Aires, Argentina) and programmed in Microsoft Excel (Microsoft, Redmond, WA). This model was developed because it includes specific characteristics of individuals, taking into account individual variability and interactions between patients, interventions, and clinical events, better representing historical and different events over time, including recurrent events, as well as the respective costs and benefits ^16^^,^^17^. This model has already been used to estimate the disease and economic burden, as well as the impact of the implementation of different tobacco control policies in other Latin American countries ^7^^,^^18^^-^^20^ and other regions ^21^. The main model compares the results (i.e., disease and economic) in a simulated cohort of Peruvians who have never smoked with those estimated by the model for Peru, according to national demographic, epidemiological, and financial parameters (Supplementary Table 1).

More information on the development and validation of the model can be found in previous publications ^9^^,^^10^^,^^18^^,^^22^. In this article, we extend the model to 2020 and consider three scenarios: neutral packaging with more than 80% of the surface area covered by health warnings, full compliance with smoke-free laws, and a complete ban on TAPS.

### Epidemiological data

The data were obtained from an extensive literature search in MEDLINE, EMBASE, Cochrane Central, SocINDEX, EconLit, LILACS, NBER, CRD, the Cochrane tobacco review team, and gray literature to find data from the Peruvian Ministry of Health, WHO data, and conference presentations. Free and controlled terms in English and Spanish related to smoking (“tobacco,” “cigarette,” “smoking”), prevalence (“prevalence,” “burden”) of each disease event, and mortality (‘mortality’) of the causes of death studied (“myocardial infarction,” “stroke,” “pneumonia,” “COPD,” “chronic obstructive pulmonary disease,” “lung cancer,” “oropharynx cancer,” “esophageal cancer,” “gastric cancer,” “pancreatic cancer,” “kidney cancer,” “laryngeal cancer,” “cervix cancer,” ”bladder cancer,“ and ”leukemia"). These terms were linked with Boolean operators and translated into each database.

We estimated the number of cases and annual mortality rates attributable to smoking, both overall and stratified by cause according to their corresponding ICD-10 codes (i.e., acute myocardial infarction [AMI] [ICD-10: I210-I229]; non-AMI coronary events [ICD-10: I200-I209]; stroke [ICD-10: I60, I61, I63, I64, I620, I621, I629, I678, I679, I690-I694, I698]; pneumonia [ICD-10: J100-J189]; chronic obstructive pulmonary disease [COPD] [ICD-10: J400-J439, J44X]; and lung cancer [ICD-10: C330-C349], oropharyngeal cancer [ICD-10: C000-C009, C140, C142, C148], esophageal cancer [ICD-10: C150-C159], stomach cancer [ICD-10: C160-C169], pancreatic cancer [ICD-10: C250-C259], renal cancer [ICD-10: C64X-C65X], laryngeal cancer [ICD-10: C320-C329], cervical cancer [ICD-10: C530-C539], bladder cancer [ICD-10: C670-C679], and leukemia [ICD-10: C920]), number of cases and percentages of disease events attributable to specific pathologies caused by smoking, and DALYs (i.e., the sum of years of life lost [YLL] and years lived with disability [YLD]) by simulating the total lifespan of each individual to obtain the total results. All were stratified by sex (i.e., males and females).

The model did not estimate the burden of perinatal effects or exposure to environmental smoke. However, we assumed that these factors contribute an additional 12% burden for women and 13.6% for men ^23^. Incidences were obtained for acute disease events, while the probabilities of developing chronic diseases were estimated using an approximation of annual mortality and survival rates from national registries. In addition, the individual risk was assessed for each event or death.

### Calibration and validation

The calibration and validation processes were carried out by comparing the specific mortality from tobacco-attributable diseases, by sex and age, predicted by the model with national statistics from Peru ^24^. Mortality data were obtained from the National Death Registry (SINADEF) ^24^ by exploring causes and disaggregation by age and sex. Mortality predictions were accepted if they were within a 15% deviation from the reference data; deviations exceeding this threshold led to adjustments in the risk equations. In addition, the model results were externally validated using independent epidemiological and clinical studies that were not involved in the initial development of the equations ^7^^,^^22^^,^^25^^-^^33^. Further details on model calibration and validation are presented in the Supplementary Material (Figures S1 and S2).

### Economic parameters

Direct medical costs

Direct medical costs (consultations, diagnosis, hospitalizations, and treatment) were extracted from previous research conducted by our team ^9^^)^ and adjusted for inflation (Peruvian inflation rate between 2015 and 2020 = 10.47%) to express all values in 2020 local currency and then converted to USD (exchange rate January 2020 = PEN 3.3) ^34^^)^ for this study. A mixed cost methodology based on national protocols, gross domestic product (GDP) per capita, and the Delphi method was used. Data were obtained from public hospitals and private clinics, adjusted for inflation to 2020 values. Further methodological details regarding the calculation of direct medical costs can be found in our previous publication ^9^^,^^18^.

Costs due to loss of labor productivity

The calculation of costs due to loss of labor productivity was based on the human capital approach, which considers two main factors: (i) the premature death of individuals and (ii) the decline in productivity at work due to health events (presenteeism). To estimate the cost of premature death, we calculated the loss of labor productivity of an individual as the present value of their future labor income, using the formula for the Value of a Statistical Life ^35^. This formula allows us to estimate productivity losses through the labor income that society loses due to the premature death of workers. Presenteeism costs are approximated as disability costs, which are measured through losses in quality of life for each health event according to the parameters shown in Table S2. Annual market wages by sex and age were estimated using a Mincer equation ^36^^,^^37^^)^ with representative national data from the 2020 National Household Survey (ENAHO) ^38^. To this end, we used the variables of labor income or wages, years of education, age, and sex from this national survey. We then applied the World Bank’s expected wage growth rate, a discount rate of 5%, and the official retirement ages in Peru by sex. For more details, see Table S1. We used an indirect estimation method based on previous research ^39^^,^^40^^)^ to estimate economic losses due to presenteeism. Specifically, we assume that the reduction in individuals’ labor productivity is directly proportional to the decline in their quality of life due to health conditions attributed to smoking ^41^. For more information and an application of this methodology, see Pinto *et al*. ^20^^)^ and Table S2.

Cost of informal care

Informal care includes unpaid hours provided by family members or friends, mainly women. Due to the lack of specific microdata on time spent on care in Peru, this study used the methodology developed by Espinola *et al*. ^42^ to estimate the time spent on informal care for patients with smoking-related diseases. For the value of the time required for informal care, hourly wage data for social and health care workers ^38^ serve as a proxy for the opportunity cost. For more details, see Espinola *et al*^42^.

Projected health and economic benefits

We studied the projected cumulative 10-year benefit on the disease burden and economic burden of implementing the following measures: 1) plain packaging with more than 80% of the surface area of packages covered by picture health warnings; 2) a total ban on TAPS; 3) full compliance with smoke-free laws; 4) and a 25%, 50%, and 75% increase in the retail price of cigarettes through taxes.

To estimate the impact of tobacco control measures, we used the methodology detailed in our previous studies ^10^. The effect was estimated on the prevalence of smoking based on the following formula:

*Prev*_*p*_ *ost =Prev*_*p*_ re - (Em * I_*p*_ * Prev_*p*_ *re)*

Where Prev_p_re is the prevalence of smokers before the intervention, Em is the effectiveness of the intervention expressed as a relative reduction in tobacco consumption, and I_p_ is the proportion of variation in consumption that impacts the prevalence of smokers. Different studies have estimated that, in the short and medium term, approximately half of the reduction in consumption is due to the reduction in prevalence and the other half is explained by the reduction in consumption among continuing smokers ^43^^-^^45^. In the case of taxes, Em represents the effect of the price change on consumption through the price elasticity of demand. The effectiveness of smoke-free interventions also considered the reduction in risk for non-smokers due to decreased exposure to secondhand smoke. The economic and health impacts were estimated by comparing the results predicted by the model for Peru with current smoking rates with those of consumption reduction after the intervention. In addition, changes in population, treatment costs, or wages used for lost productivity were not considered. Therefore, the estimated savings correspond to a steady state where only changes in prevalence due to the policy occur. The impact of the interventions is reported as a cumulative effect over 10 years.

### Ethics

This study did not require ethical approval because it used public databases and articles published during the development of the model.

## RESULTS

We estimate that approximately 22,350 deaths are attributable to tobacco use in Peru, representing approximately 22.4% of the country’s total annual mortality and 37.1% of total deaths from smoking-related diseases in people over 35 years of age. Approximately 19% of all deaths from heart disease and 18% of deaths from stroke can be attributed to smoking. The specific attributable percentages for each disease are highest for laryngeal cancer (84.6%), lung cancer (83.2%), and COPD (80.1%), even when stratified by sex. In addition, 21% of deaths from pneumonia can be attributed to smoking, and 2,574 deaths are attributed to passive smoking (Table 1).

**Table 1:** Annual disease burden attributable to tobacco consumption in Peru, 2020.

Condition	Total deaths	Deaths attributable to tobacco use						Total disease events	Disease events attributable to tobacco use					
		Total		Men		Women			Total		Men		Women	
		n	%	n	%	n	%		n	%	n	%	n	%
Acute myocardial infarction	6348	1303	20.5	841	23.4	462	16.8	20716	5063	24.4	3700	27.6	1363	18.7
Ischemic heart disease	794	189	23.9	121	25.8	68	21.1	12902	3575	27.7	2280	29.2	1295	25.4
Non-ischemic heart disease*	4599	759	16.5	530	22	228	10	-	-	-	-	-	-	-
Stroke	8288	1538	18.6	912	22.5	626	14.8	53016	10655	20.1	6006	23.6	4649	16.9
Lung cancer	2910	2420	83.2	1324	89.7	1096	76.5	3294	2730	82.9	1455	89.6	1275	76.4
Pneumonia	13719	2874	21	1684	24.2	1190	17.6	110662	24169	21.8	13385	24.6	10784	19.2
COPD	9517	7625	80.1	3910	81.5	3715	78.7	103633	74959	72.3	39419	74.2	35540	70.4
Oropharyngeal cancer	565	368	65.1	231	78.1	137	50.8	1194	777	65.1	472	77.1	305	52.4
Esophageal cancer	366	260	71.3	186	73.5	74	66.3	461	325	70.6	232	73.2	93	64.8
Stomach cancer	4692	1041	22.2	749	29.5	292	13.6	5877	1314	22.4	951	29.6	363	13.6
Pancreatic cancer	1561	457	29.3	242	30.2	215	28.4	1808	530	29.3	281	30.2	249	28.4
Kidney cancer	808	237	29.4	221	41.5	16	6	1518	452	29.7	416	42.1	36	6.8
Laryngeal cancer	136	115	84.6	86	85.7	29	81.1	286	234	81.8	177	84.2	57	75
Leukemia	1204	209	17.4	153	25	56	9.4	1742	308	17.7	224	25.3	84	9.8
Bladder cancer	384	165	43	122	49.4	43	31.3	910	397	43.6	302	49.2	95	32.1
Cervical cancer	1811	236	13	NA	NA	236	13	3819	508	13.3	NA	NA	508	13.3
Exposure to environmental smoke and other causes	2574	2557	100	1539	100	1018	100	NA	NA	NA	NA	NA	NA	NA
Total	60275	22353	37.1	12581	41.9	9502	32,1	321840	125996	39.1	69300	-	56696	-

n: absolute frequency; COPD: chronic obstructive pulmonary disease; NA: not applicable.

*No tobacco-attributable disease events are presented for non-ischemic heart disease due to the lack of relative measures that allow for their calculation in the consulted data sources.

Note: The percentage of condition-specific deaths is the proportion of deaths attributable to tobacco use out of the total condition-specific deaths in the population aged 35 years and older (e.g., deaths caused by acute myocardial infarction in males: 841/23.4% means that there are 841 deaths from acute myocardial infarction attributable to smoking in men, representing 23.4% of all deaths from that condition in men). The percentage of condition-specific disease events is the proportion of events attributable to tobacco use out of the total number of condition-specific events (e.g., events caused by acute myocardial infarction in males: 3,700/27.6% means that there are 3,700 acute myocardial infarction events in men attributable to smoking, representing 27.6% of the total events for that condition in men). The results were obtained from the model developed using the parameters shown in Table S1 of the supplementary material, which were obtained after the literature search mentioned in the Epidemiological Data subsection.

In addition, we estimate a total of 126,000 annual disease events attributable to smoking (39.1% of all smoking-related diseases). Among men and women, the most frequent disease events attributed to smoking were lung cancer (89.6% and 76.4%), laryngeal cancer (84.2% and 75%), oropharyngeal cancer (77.1% and 52.6%), and COPD (74.2% and 70.4%) (Table 1).

### Life expectancy and quality of life associated with smoking

Life expectancy for male smokers was 6.5 years shorter than for non-smokers, and for male ex-smokers it was 3.2 years shorter. Among women, smokers had a life expectancy 7.5 years shorter than non-smokers, and ex-smokers experienced a reduction of 3.1 years. Thus, we estimated that 715,158 DALYs, with 515,768 YLLs and 199,801 YLDs (107,948 for men and 91,853 for women) are attributable to smoking annually in Peru.

### Costs associated with smoking

**Figure 1:**
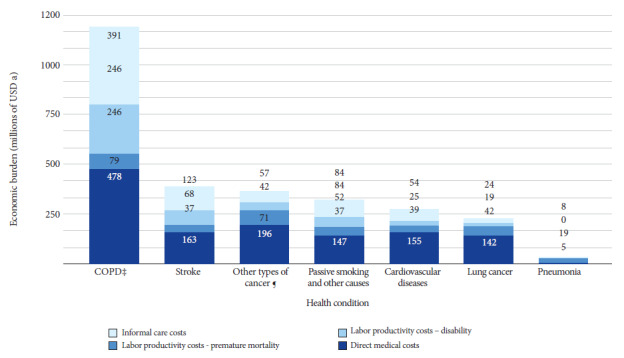
Annual economic burden attributable to tobacco consumption in Peru by condition and type of expenditure (millions of USD a), 2020.

‡COPD: Chronic obstructive pulmonary disease; §Cardiovascular diseases: Acute myocardial infarction, ischemic heart disease, non-myocardial infarction, non-ischemic cardiovascular disease. ¶Other types of cancer: Oral, stomach, esophagus, pancreas, kidney, bladder, larynx, cervix, and leukemia. ^a^ Monetary values are expressed in 2020 USD. Exchange rate January 2020: USD 1 = PEN 3.3 (Soles)

The estimated economic burden of smoking in Peru is approximately USD 2804 billion, with USD 1285 billion attributed to direct medical costs (45.8%), USD 453 million to productivity losses due to disability (16.2%), USD 325 million to premature death (11.6%), and USD 741 million to informal care costs (26.4%). COPD represented the highest cost (USD 1194 billion), followed by stroke (USD 391 million) and other types of cancer (USD 365 million). The estimated economic burden represented 1.28% of gross domestic product, and the direct cost attributed to tobacco use represented 0.59% of GDP (Table 2 and Figure 1).

**Table 2:** Annual economic burden attributable to tobacco consumption in Peru by sex, health status, and type of costs, 2020.

Cost type		Sex	Attributable costs (millions of US dollars)							
			COPD	Cardiovascular diseases	Lung cancer	Other types of cancer	Passive smoking and other causes	Stroke	Pneumonia	Total
Direct medical costs		Men	231.0	48.3	71.1	73.7	60.3	75.9	2.1	562.4
		Women	247.1	107.0	70.6	122.0	86.5	86.9	2.6	722.7
		Total	478.0	155.3	141.7	195.8	146.8	162.9	4.8	1285.2
Costs of lost productivity	Premature mortality	Men	46.3	30.9	24.3	45.9	25.1	23.4	13.6	209.5
		Women	32.5	8.1	18.1	25.0	12.4	13.8	5.7	115.6
		Total	78.8	39.1	42.4	70.8	37.5	37.3	19.3	325.1
	Disability	Men	144.6	20.2	10.7	27.9	32.9	38.1	0.1	274.5
		Women	101.8	5.2	8.5	13.7	19.1	30.2	0.0	178.5
		Total	246.3	25.4	19.2	41.6	52.0	68.3	0.1	453.0
Informal care costs		Men	201.4	36.8	12.3	35.7	48.2	64.0	4.4	402.9
		Women	189.2	16.7	12.2	21.0	36.2	58.6	3.6	337.6
		Total	390.6	53.6	24.5	56.8	84.4	122.6	8.0	740.5
Total			1193.8	273.3	227.7	365.0	320.6	391.1	32.2	2803.7

COPD: Chronic obstructive pulmonary disease.

Monetary values are expressed in 2020 USD. Exchange rate in January 2020: USD 1 = PEN 3.3 (Soles).

### Expected benefits of strengthening tobacco control measures

In ten years, plain packaging would prevent 6,218 deaths, 31,700 events, 200,900 YLD, and USD 576 million in costs attributed to tobacco use. In addition, full compliance with smoke-free laws would prevent 4,982 deaths, 25,400 events, 160,959 DALYs, and USD 461 million in expenditures attributed to tobacco use. Likewise, a total ban on TAPS would prevent 8,767 deaths, 44,700 events, 283,270 DALYs, and USD 812 million in expenditures attributed to tobacco use. Most of the deaths and events prevented in the three scenarios occurred in COPD and pneumonia attributable to tobacco use, while cost reductions were mainly found in COPD and stroke (Table 3).

**Table 3:** Projected ten-year scenarios for mortality reduction, events, and associated costs with strengthened tobacco control measures, 2020-2029.

Scenarios		Reduction in mortality		Reduction in events		Cost reduction (millions of US dollars ^a^)	
		Base case (Ll - UL)	Estimated reduction (Ll - UL)	Base case (Ll - UL)	Estimated reduction (Ll - UL)	Base case (Ll - UL)	Estimated reduction (Ll - UL)
Neutral packaging and more than 80% of the surface area covered with health warnings							
	Cardiovascular diseases	646 (323-970)	688 (361-1742)	2525 (1263-3788)	2687 (1410-6806)	62.82 (31.41-94.24)	66.83 (35.09-169.31)
	Stroke	670 (335-1005)	712 (374-1805)	4606 (2303-6909)	4900 (2573-12414)	123.38 (61.69-185.07)	131.25 (68.91-332.51)
	COPD	1539 (769-2308)	1637 (859-4147)	13202 (6601-19803)	14044 (7373-35579)	177.39 (88.69-266.08)	188.71 (99.07-478.06)
	Pneumonia	861 (430-1291)	915 (481-2319)	7461 (3731-11192)	7937 (4167-20108)	3.94 (1.97-5.91)	4.19 (2.2-10.62)
	Lung cancer	678 (339-1017)	721 (379-1827)	762 (381-1144)	811 (426-2055)	46.49 (23.25-69.74)	49.46 (25.97-125.3)
	Other types of cancer	779 (389-1168)	829 (435-2099)	1247 (624-1871)	1327 (696-3361)	65.54 (32.77-98.31)	69.72 (36.6-176.63)
	Exposure to environmental smoke and other causes	672 (336-1009)	715 (376-1812)	NA	NA	61.83 (30.92-92.75)	65.78 (34.54-166.65)
	Total	5845 (2922-8767)	6218 (3265-15751)	29804 (14902-44706)	31706 (16645-80323)	541.4 (270.7-812.1)	575.96 (302.38-1459.09)
Full compliance with anti-smoking laws							
	Cardiovascular diseases	1129 (572-1671)	551 (236-1052)	3788 (2104-9891)	2152 (922-4108)	109.76 (55.62-162.41)	53.55 (22.94-102.2)
	Stroke	1170 (593-1731)	571 (245-1089)	6909 (3838-18041)	3926 (1682-7493)	215.56 (109.24-318.96)	105.16 (45.05-200.7)
	COPD	2689 (1363-3978)	1312 (562-2503)	19803 (11001-51707)	11252 (4820-21475)	309.91 (157.05-458.57)	151.19 (64.76-288.55)
	Pneumonia	1503 (762-2225)	733 (314-1400)	11192 (6218-29223)	6359 (2724-12137)	6.89 (3.49-10.19)	3.36 (1.44-6.41)
	Lung cancer	1184 (600-1752)	578 (247-1103)	1144 (635-2986)	650 (278-1240)	81.23 (41.17-120.2)	39.63 (16.98-75.63)
	Other types of cancer	1361 (690-2014)	664 (284-1267)	1871 (1039-4884)	1063 (455-2029)	114.51 (58.03-169.43)	55.86 (23.93-106.61)
	Exposure to environmental smoke and other causes	1175 (595-1738)	573 (246-1094)	NA	NA	108.03 (54.75-159.85)	52.7 (22.58-100.58)
	Total	10212 (5175-15110)	4982 (2134-9508)	44706 (24836-116731)	25402 (10881-48482)	945.88 (479.34-1399.62)	461.45 (197.67-880.68)
Total ban on TAPS ^b^							
	Cardiovascular diseases	108 (0-1465)	970 (539-2532)	421 (0-5724)	3788 (2104-9891)	10.47 (0-142.4)	94.24 (52.35-246.06)
	Stroke	112 (0-1518)	1005 (558-2623)	768 (0-10441)	6909 (3838-18041)	20.56 (0-279.66)	185.07 (102.82-483.23)
	COPD	256 (0-3488)	2308 (1282-6027)	2200 (0-29924)	19803 (11001-51707)	29.56 (0-402.07)	266.08 (147.82-694.76)
	Pneumonia	143 (0-1951)	1291 (717-3370)	1244 (0-16912)	11192 (6218-29223)	0.66 (0-8.93)	5.91 (3.28-15.44)
	Lung cancer	113 (0-1537)	1017 (565-2655)	127 (0-1728)	1144 (635-2986)	7.75 (0-105.39)	69.74 (38.75-182.1)
	Other types of cancer	130 (0-1766)	1168 (649-3051)	208 (0-2827)	1871 (1039-4884)	10.92 (0-148.56)	98.31 (54.62-256.7)
	Exposure to environmental smoke and other causes	112 (0-1524)	1009 (560-2634)	NA	NA	10.31 (0-140.16)	92.75 (51.53-242.19)
	Total	974 (0-13248)	8767 (4871-22892)	4967 (0-67555)	44707 (24835-116732)	90.23 (0-1227.17)	812.1 (451.17-2120.48)

Ll: lower limit; UL: upper limit; COPD: chronic obstructive pulmonary disease; NA: not applicable; TAPS: tobacco advertising, promotion, and sponsorship

a Monetary values are expressed in 2020 USD. Exchange rate January 2020: USD 1 = PEN 3.3 (Soles)

b The base case corresponds to a comprehensive ban on TAPS and the estimated reduction corresponds to a complete ban on TAPS.

**Figure 2:**
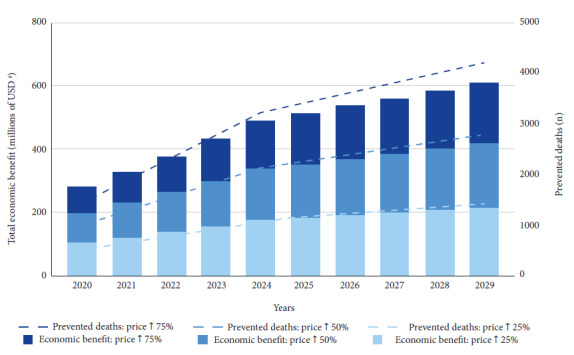
Comparative analysis of the projected impact of three different scenarios of cigarette price increases, both in terms of estimated number of deaths prevented and economic cost savings over ten years in Peru (millions of US dollars), 2020-2029.

In addition, approximately 10,180, 20,400, and 30,500 deaths and 329,200, 658,400, and 987,700 years of healthy life would be avoided through a 25%, 50%, and 75% increase in cigarette prices. The three price increase scenarios would save USD 1717 million, USD 3300 million, and USD 4752 million over ten years, respectively (Table 4 and Figure 2).

**Table 4:** Cumulative economic and health benefits over 10 years from a 25%, 50%, and 75% increase in the retail price of cigarettes through taxes in Peru, 2020-2029.

Benefits		Base case Price increase		
		25%	50%	75%
Health effects (n)				
	Prevented deaths	10180	20359	30539
	Prevented healthy life years lost	329221	658443	987664
	Prevented coronary heart disease events	4398	8797	13195
	Prevented stroke events	8022	16045	24067
	Prevented COPD events	22993	45986	68979
	Prevented cancer events	3500	7000	10499
Economic effects (millions of USD ^a^)				
	Savings in healthcare costs	600	1201	1801
	Savings in costs due to lost productivity	336	672	1007
	Cost savings for informal caregivers	343	685	1028
	Increase in tax revenue	438	743	915
Total economic benefit		1717	3300	4752

a Monetary values are expressed in USD for 2020. January 2020 exchange rate: USD 1 = PEN 3.3 (Soles)

n: absolute frequency ^a^ Monetary values are expressed in USD as of 2020. Exchange rate as of January 2020: USD 1 = PEN 3.3 (Soles)

## DISCUSSION

According to our results, approximately 22,350 Peruvians die each year due to tobacco use, representing more than 22.4% of total annual deaths in Peru. In addition, 126,000 events were attributable to tobacco use, with a high burden due to COPD, lung cancer, pneumonia, a total loss of more than 515,000 years of life lost, and more than 199,000 years of healthy life lost. Furthermore, the economic burden attributable to tobacco consumption reached USD 2804 billion, approximately 1.28% of GDP in 2020.

In our model, each tobacco control measure contributed to a significant reduction in deaths (between 4,900 and 8,700), events (between 25,400 and 44,700), and attributable costs (between US$461 million and US$812 million) attributable to tobacco use. A 50% increase scenario significantly reduced deaths (20,300 deaths) and increased the number of healthy life years lost avoided (658,000 years) over a ten-year period if current benefits are maintained.

In Latin America, 350,593 deaths and 2,248,394 events per year were attributed to tobacco use in 2020 among twelve countries ^7^. Brazil and Mexico topped this list, while Peru ranked fifth ^7^. In addition, in 2016, we estimated the disease burden of tobacco use in Peru at 16,719 deaths per year and 95,665 events per year attributable to smoking, representing 12.5% of total deaths, less than our current estimate ^9^. This could be due to higher prevalence rates of diseases attributable to tobacco use, such as COPD (a 24.6% increase in age-standardized prevalence rates) ^46^ and lung cancer (an absolute increase of 93%) ^47^^)^ reported from 1990 to 2019, due to better and earlier detection, efforts to increase early detection of other tobacco-related diseases, and the simultaneous influence of other risk factors such as exposure to biomass and tuberculosis, among others ^48^.

The total economic burden attributed to tobacco in Latin American countries was USD 49.804 billion, with Brazil at the top of the list, followed by Mexico, while Peru ranked fifth ^7^. Our 2016 estimate calculated an annual economic burden in Peru of PEN 2.535 billion, representing 0.4% of GDP ^9^. However, this figure did not include indirect financial costs. The current estimate shows an increase to USD 2.803 billion, with indirect costs comprising 52.7% of the total economic burden, representing a greater share of Peru’s GDP (1.28%). Respiratory diseases and cancers lead the financial burden, followed by cerebrovascular accidents. On the other hand, passive smoking and other causes contributed USD 320 million (11.4%), most of which came from direct costs and informal care. Similar results were reported for Argentina, Brazil, Chile, Colombia, and Ecuador ^7^, highlighting the need to discuss the social costs attributable to tobacco during policy discussions, such as the disproportionate impact on women due to their role in informal care ^49^.

Although Peru has taken necessary measures to control tobacco in recent decades ^(11-13)^, it still faces challenges in reducing its economic and health impact. According to our model, the most effective strategies include stricter regulation of the ban on TAPS, an increase in cigarette taxes to reduce accessibility, and greater adherence to smoke-free laws ^13^. In addition, plain packaging with more than 80% of the surface area covered by health warnings also contributed to a reduction in deaths, events, and costs attributable to smoking. These findings are consistent with studies from other Latin American countries ^18^^,^^50^, in which tobacco control measures projected a considerable reduction in the economic and disease burden. This shows that Latin American countries share a significant gap for improvement that would lead to favorable results if addressed with stricter measures ^18^^,^^50^.

Although Peru has achieved an adequate level of warning about the dangers of tobacco, it does not yet fully comply with TAPS bans and smoke-free laws, scoring eight out of ten points in these areas ^13^. To achieve full compliance, an explicit and comprehensive ban on smoking in outdoor public places is required. According to the WHO, regulation should focus on universities, outdoor areas, private vehicles with children, outdoor playgrounds, bars, and pubs, where Peru scored between three and five points out of a total of ten ^13^. To strengthen these measures, it is recommended to set up telephone numbers or other mechanisms to report violations, impose fines on establishments that do not remove ashtrays, allocate funds for the enforcement of regulations, and explicitly ban heated tobacco products and nicotine devices, both electronic and non-electronic ^13^.

Considering that increasing taxes on cigarettes is the most cost-effective measure to reduce tobacco consumption ^51^, our model showed that a 50% price increase would significantly reduce deaths and healthy life years lost over ten years. Our results indicate that higher price increases have a greater impact on reducing consumption and associated mortality, with a 75% increase producing the greatest economic benefits, which are amplified over time. This suggests that the positive effects of a price increase are not only immediate but accumulate over the years. Although Peru has made remarkable progress by increasing cigarette taxes to 73.3%, it has not yet reached the 75% recommended by the WHO and must adjust the tax in line with inflation to avoid a real reduction in price ^13^. Despite updating its tobacco tax in January 2024 ^52^, Peru has not achieved this goal yet.

Our results should be interpreted in light of some limitations. The model does not include the impact of alternative tobacco products, such as electronic cigarettes and heated tobacco, which could underestimate the actual burden of the nicotine epidemic in Peru. It should be noted that electronic nicotine delivery systems or nicotine-free systems were not regulated in the country during the study period. Furthermore, healthcare costs were estimated based on expert information, local clinical practice guidelines, and specialized literature, which may not reflect regional differences in diagnosis, treatment, and follow-up, affecting the accuracy of healthcare cost estimates. In addition, the model represents national-level results, and local or regional variations are likely to exist.

In addition, several costs outside the health sector were not included, such as the cost of labor productivity due to absenteeism or the environmental impact. Furthermore, considering economic growth, productivity gains, and demographic changes could improve the accuracy of the 10-year projections. Nevertheless, the findings remain valuable, as they can be interpreted as a steady-state scenario or as the present value of benefits over the decade. We also did not take into account equity factors when increasing tobacco taxes on the population, which could be better explored in a distributional cost-effectiveness analysis or an extended cost-effectiveness analysis. Furthermore, we did not consider the impact of the COVID-19 pandemic during 2020 on smoking prevalence and secondhand smoke exposure due to mobility restrictions ^53^, as well as other health and socioeconomic disruptions that occurred during this period ^54^. Furthermore, tobacco control measures were analyzed in isolation, although there is evidence that their joint implementation would generate synergistic effects, which could enhance the estimated benefits ^55^. Finally, no systematic review was conducted to search for the information included in the model; however, the literature search was exhaustive and covered different databases, including the best evidence available at the time the model was developed.

On the other hand, our study also has significant strengths. Given that specific data on policy implementation are difficult to obtain from prospective studies, our model represents a significant opportunity to simulate the impact of tobacco control measures, providing policymakers in Peru with data for the decision-making process. In addition, this study represents the most comprehensive and up-to-date report that adds indirect costs to the economic burden.

In conclusion, the disease and economic burden of tobacco use in Peru remains considerable. To mitigate its impact, it is crucial to implement higher taxes on cigarettes, ensure full compliance with smoke-free regulations, introduce plain packaging, and impose a comprehensive ban on tobacco advertising, promotion, and sponsorship. These measures have the potential to significantly reduce both the prevalence of smoking and the associated health and economic costs. Therefore, prioritizing these policy interventions is essential to advance public health and economic sustainability in Peru.
