# Supplementary material for: Disease burden from tobacco consumption in Peru and the projected effect of strengthening control measures: a modeling study
Source: Rev Peru Med Exp Salud Publica. 2025 Jun 23;42(2):126–37. doi: 10.17843/rpmesp.2025.422.14338 (PMC12377892; doi:10.17843/rpmesp.2025.422.14338)
Supplement: Supplementary material. — Available in the electronic version of the RPMESP. [file rpmesp-42-02-14338-s001.docx]

**Material suplementario**

# Tabla S1. Principales parámetros demográficos, epidemiológicos y económicos considerados en el modelo (2020).

| **Parámetro** | | **Valor** | **Fuente** |
| --- | --- | --- | --- |
| **Población 2020**  **(> 35 años)** | Total | 13,390,445 | [^(1)^](https://paperpile.com/c/uKBKkR/uax1b) |
|  | Varones > 35 años | 49% | [^(1)^](https://paperpile.com/c/uKBKkR/uax1b) |
|  | Mujeres > 35 años | 51% | [^(1)^](https://paperpile.com/c/uKBKkR/uax1b) |
| **Prevalencia de tabaquismo** | Men > 35 años | 29% | [^(1)^](https://paperpile.com/c/uKBKkR/uax1b) |
|  | Women > 35 años | 18% | [^(1)^](https://paperpile.com/c/uKBKkR/uax1b) |
| **Mortalidad cruda general**  **tasa de mortalidad por 100,000**  **(Varones/Mujeres)** | Infarto agudo de miocardio | 45,5  (52,5/38,8) | [^(2)^](https://paperpile.com/c/uKBKkR/gJkAK) |
|  | Enfermedad cardíaca isquémica  (enfermedad cardíaca no isquémica) | 5,4  (6,5/4,3) | [^(2)^](https://paperpile.com/c/uKBKkR/gJkAK) |
|  | Otras enfermedades cardiovasculares | 32,8  (35/30,6) | [^(2)^](https://paperpile.com/c/uKBKkR/gJkAK) |
|  | Accidente cerebrovascular | 55,8  (55,6/56,1) | [^(2)^](https://paperpile.com/c/uKBKkR/gJkAK) |
|  | Neumonía/Influenza | 98,8 (101,9/95,9) | [^(2)^](https://paperpile.com/c/uKBKkR/gJkAK) |
|  | Cáncer de pulmón | 20,6  (21,2/20) | [^(2)^](https://paperpile.com/c/uKBKkR/gJkAK) |
| **Costos médicos directos 2020 (USD)** | Evento de infarto agudo de miocardio | 2.979 | Estimación propia[^(3)^](https://paperpile.com/c/uKBKkR/kIeS5) |
|  | Evento de enfermedad cardíaca no isquémica | 2.070 |  |
|  | Enfermedad cardíaca coronaria (seguimiento anual) | 1.310 |  |
|  | Evento de accidente cerebrovascular | 5.660 |  |
|  | Accidente cerebrovascular (seguimiento anual) | 898 |  |
|  | Neumonía/Influenza | 195 |  |
|  | EPOC leve (anual) | 163 |  |
|  | EPOC moderado (anual) | 427 |  |
|  | EPOC severo (anual) | 4.882 |  |
|  | Cáncer de pulmón: 1er año | 15.756 |  |
|  | Cáncer de pulmón: 2ndo año | 20.373 |  |
| **Productividad laboral y costos del cuidado informal** | Salario horario promedio cuidador | 2,35 | ENAHO 2020[^(4)^](https://paperpile.com/c/uKBKkR/mHklT) |
| **Salario anual (promedio)** | Varones | 7.430,65 |  |
|  | Mujeres | 5.816,79 |  |
|  | Varones 35-49 años | 7.604,61 |  |
|  | Mujeres 35-49 años | 6.139,43 |  |
|  | Varones 50-65 años | 7.257,26 |  |
|  | Mujeres 50-65 años | 5.470,32 |  |
| **Crecimiento salarial real esperado** | | 1,64% | [^(5)^](https://paperpile.com/c/uKBKkR/PwXwt) |
| **Edad de jubilación** | Varones | 65 | [^(6)^](https://paperpile.com/c/uKBKkR/guLlB) |
|  | Mujeres | 65 | [^(6)^](https://paperpile.com/c/uKBKkR/guLlB) |
| **Parámetros económicos** | Elasticidad precio de la demanda | -0,42  (-0,5 to 0,3) | [^(7)^](https://paperpile.com/c/uKBKkR/nBZjO) |
|  | Ingresos por impuestos al tabaco en 2020 (millones de USD) | 476,47 | [^(8)^](https://paperpile.com/c/uKBKkR/ku1cR) |
|  | Impuestos como proporción del precio de los cigarrillos | 49% | [^(8)^](https://paperpile.com/c/uKBKkR/ku1cR) |
|  | PBI en 2020 (millones de USD) | 201.409 | [^(5)^](https://paperpile.com/c/uKBKkR/PwXwt) |
|  | Gasto total en salud (proporción del PBI) | 6,29% | [^(5)^](https://paperpile.com/c/uKBKkR/PwXwt) |

Los valores monetarios se expresan en USD de 2020. Tipo de cambio enero 2020: USD 1 = PEN 3,3 (soles peruanos).

EPOC: Enfermedad pulmonar obstructiva crónica. PBI: Producto bruto interno.

# Tabla **S2.** Años de vida ajustados por calidad (AVAC) para cada evento.

| **Evento** | **AVAC** | **Fuente** |
| --- | --- | --- |
| Enfermedad coronaria crónica | 0,85 | [^(9)^](https://paperpile.com/c/uKBKkR/i5J3V) |
| Infarto agudo de miocardio | 0,803 | [^(10)^](https://paperpile.com/c/uKBKkR/yqsQD) |
| EPOC leve | 0,935 | [^(11)^](https://paperpile.com/c/uKBKkR/SDp4W) |
| EPOC moderado | 0,776 | [^(11)^](https://paperpile.com/c/uKBKkR/SDp4W) |
| EPOC severo | 0,689 | [^(11)^](https://paperpile.com/c/uKBKkR/SDp4W) |
| Accidente cerebrovascular | 0,62 | [^(12)^](https://paperpile.com/c/uKBKkR/SZKHJ) |
| Seguimiento de accidente cerebrovascular | 0,78 | [^(12)^](https://paperpile.com/c/uKBKkR/SZKHJ) |
| Evento coronario que no fue infarto agudo de miocardio | 0,803 | [^(10)^](https://paperpile.com/c/uKBKkR/yqsQD) |
| Neumonía | 0,994 | [^(13)^](https://paperpile.com/c/uKBKkR/xbtwc) |
| Cáncer de pulmón | 0,66 | [^(14)^](https://paperpile.com/c/uKBKkR/9r3Wb) |
| Cáncer de boca | 0,745 | [^(15)^](https://paperpile.com/c/uKBKkR/31h01) |
| Cáncer esofágico | 0,63 | [^(16)^](https://paperpile.com/c/uKBKkR/Q1eS1) |
| Cáncer gástrico | 0,55 | [^(17)^](https://paperpile.com/c/uKBKkR/ZwxEy) |
| Cáncer pancreático | 0,55 | [^(18)^](https://paperpile.com/c/uKBKkR/r2X2D) |
| Cáncer renal | 0,78 | [^(19)^](https://paperpile.com/c/uKBKkR/txMMW) |
| Cáncer laríngeo | 0,76 | [^(19)^](https://paperpile.com/c/uKBKkR/txMMW) |
| Leucemia | 0,82 | [^(20)^](https://paperpile.com/c/uKBKkR/Eykuy) |
| Cáncer vesical | 0,678 | [^(21)^](https://paperpile.com/c/uKBKkR/yB29G) |
| Cáncer de cérvix | 0,758 | [^(22)^](https://paperpile.com/c/uKBKkR/TkVW6) |

AVAC: años de vida ajustados por calidad, EPOC: Enfermedad pulmonar obstructiva crónica.

# Figura **S1.** Calibración del modelo, número anual de muertes predichas por el modelo en comparación con las estadísticas nacionales[^(1,2)^](https://paperpile.com/c/uKBKkR/uax1b+gJkAK) y Globocan[^(23)^](https://paperpile.com/c/uKBKkR/OsRnB) en cuatro condiciones seleccionadas

1. b)

**
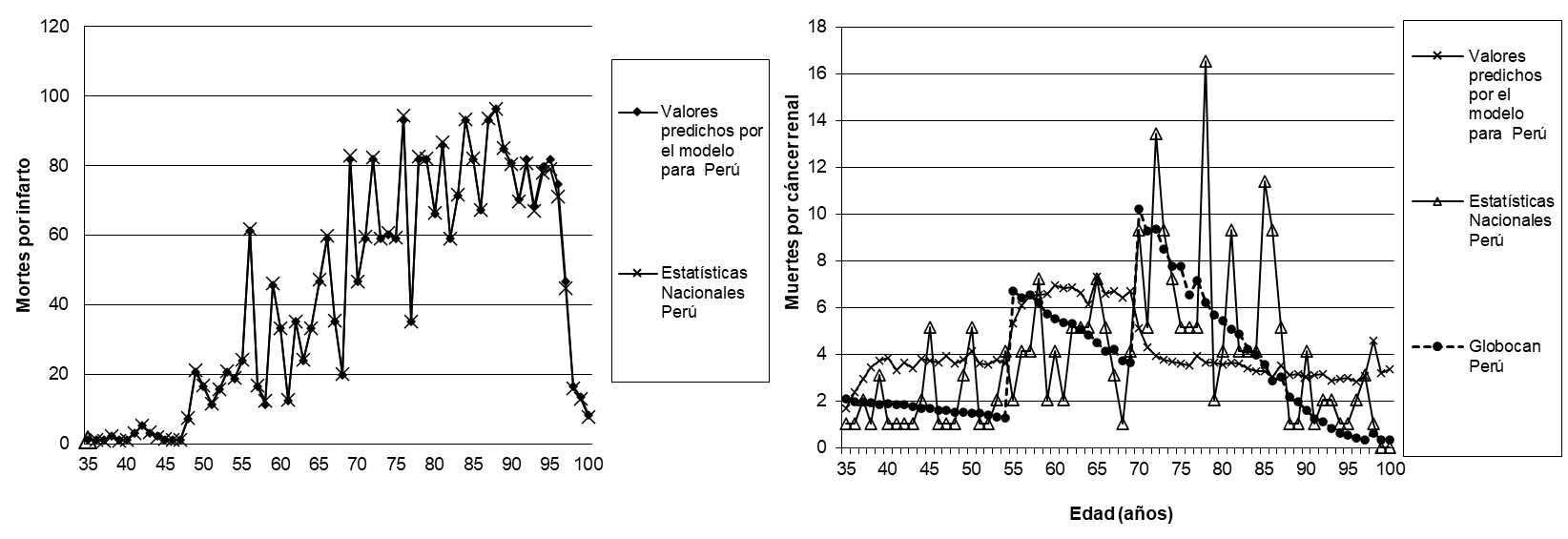
**

c) d)

**
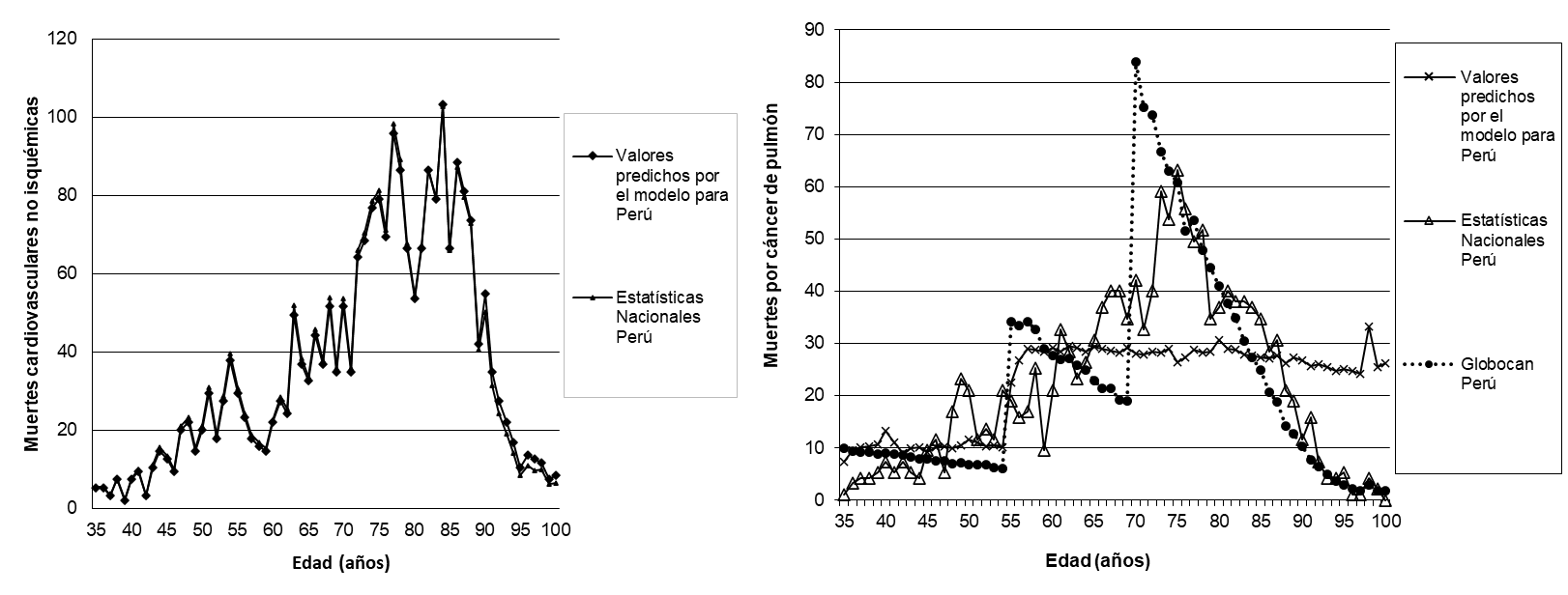
**

| (a) Infarto de miocardio (mujeres); (b) Cáncer de riñón (mujeres); (c) Muertes cardiovasculares no isquémicas (hombres); y (d) Cáncer de pulmón (hombres) Figura **S2.** Validación del modelo con estudios epidemiológicos seleccionados, resultados correspondientes a la población masculina.  1. b) | | | | | | | | | | | |
| --- | --- | --- | --- | --- | --- | --- | --- | --- | --- | --- | --- |
|  |  |  |  |  |  |  |  |  |  |  |  |
|  |  |  |  |  |  |  |  |  |  |  |  |
|  |  |  |  |  |  |  |  |  |  |  |  |
|  |  |  |  |  |  |  |  |  |  |  |  |
|  |  |  |  |  |  |  |  |  |  |  |  |


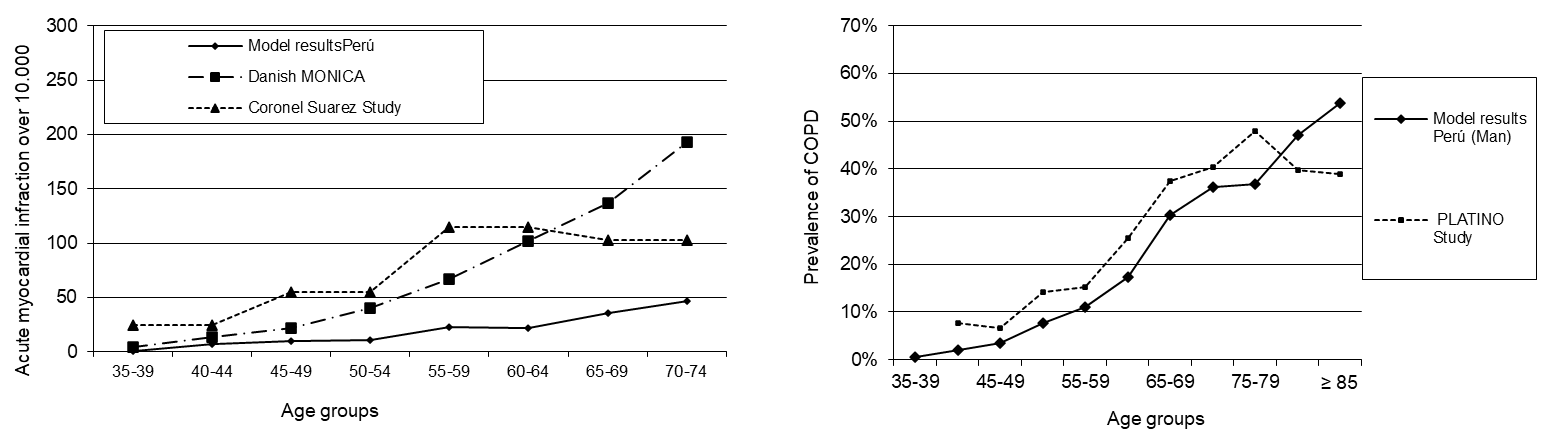


c) d)


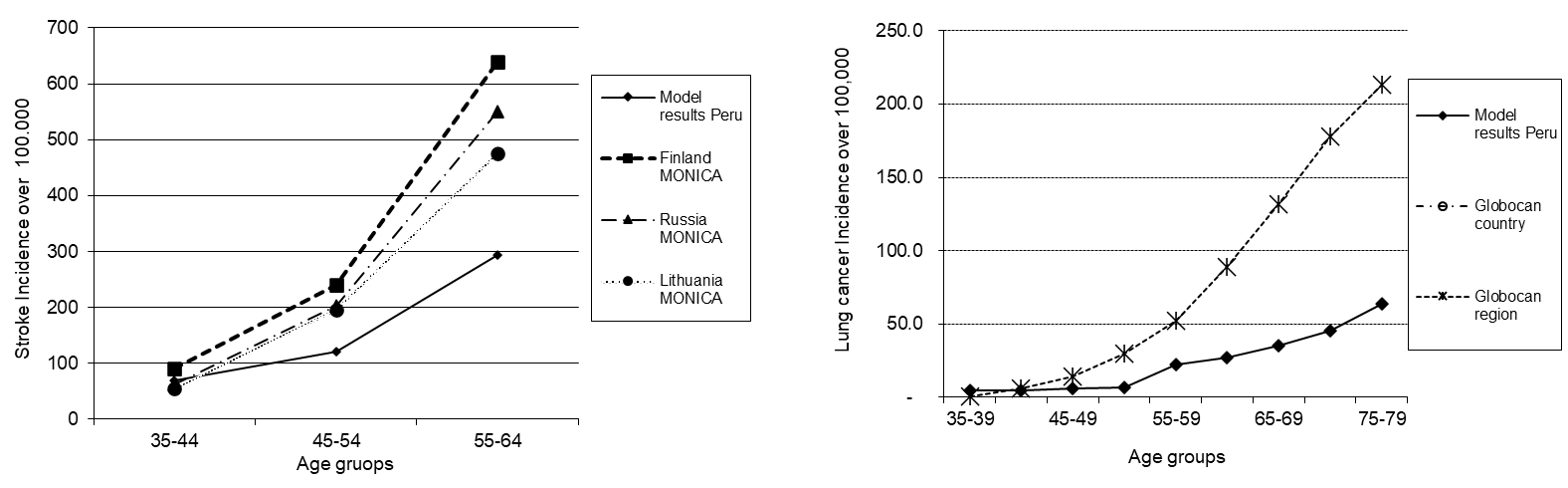


e) f)

**
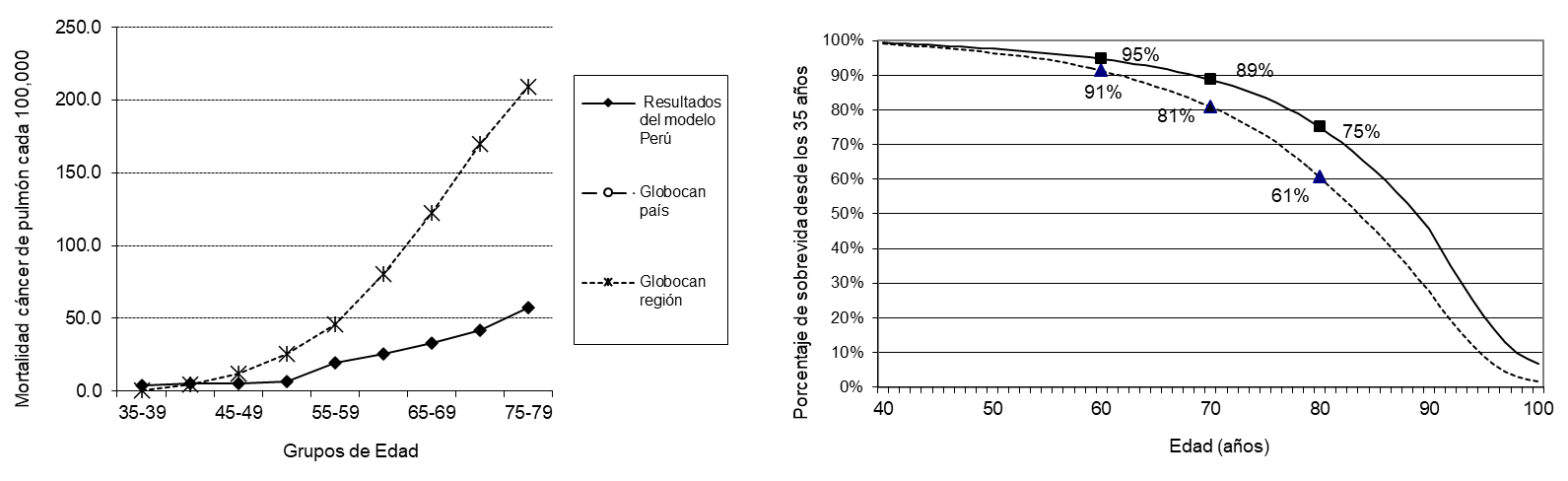
**

(a) Incidencia de accidente cerebrovascular predicha por el modelo comparada con estudios poblacionales de incidencia: registro del estudio MONICA de la OMS de Dinamarca[^(24,25)^](https://paperpile.com/c/uKBKkR/uDZdl+ki4xR) y estudio de incidencia de infarto en Argentina (Coronel Suárez)[^(26)^](https://paperpile.com/c/uKBKkR/Wq0wS); (b) Prevalencia de EPOC predicha por el modelo comparada con la prevalencia informada por el estudio PLATINO (Proyecto Latinoamericano PLATINO para la Investigación de la Enfermedad Pulmonar Obstructiva)[^(27)^](https://paperpile.com/c/uKBKkR/29ojm); (c) Incidencia de accidente cerebrovascular predicha por el modelo comparada con el registro del estudio MONICA de la OMS en países seleccionados (registro del estudio MONICA de la OMS de Finlandia, provincia de Karelia del Norte[^(28)^](https://paperpile.com/c/uKBKkR/rAMaf), registro del estudio MONICA de la OMS de Rusia, ciudad de Novosibirsk[^(29)^](https://paperpile.com/c/uKBKkR/MYXeO), registro del estudio MONICA de la OMS de Lituania, ciudad de Kaunas)[^(30)^](https://paperpile.com/c/uKBKkR/Vqp3m); (d) Incidencia de cáncer de pulmón predicha por el modelo comparada con las estimaciones de la Agencia Internacional para la Investigación sobre el Cáncer (IARC)[^(23)^](https://paperpile.com/c/uKBKkR/OsRnB); (e) Tasa de mortalidad por cáncer de pulmón predicha por el modelo comparada con las estimaciones de la IARC[^(23)^](https://paperpile.com/c/uKBKkR/OsRnB); f) Supervivencia a partir de los 35 años en fumadores y no fumadores, resultados predichos por el modelo[^(31)^](https://paperpile.com/c/uKBKkR/8qKUS).

# Referencias

1. [INEI. Estimaciones y Proyecciones de Población [Internet]. Instituto Nacional de Estadística e informática. [citado el 22 de julio de 2024]. Disponible en:](http://paperpile.com/b/uKBKkR/uax1b) <https://www.inei.gob.pe/estadisticas/indice-tematico/population-estimates-and-projections/>

2. [Ministerio de Salud de Perú. Información de Fallecidos del Sistema Informático Nacional de Defunciones - SINADEF [Internet]. SINADEF. [citado 2022]. Disponible en:](http://paperpile.com/b/uKBKkR/gJkAK) [https://www.datosabiertos.gob.pe/dataset/información-defallecidos-del-sistema-informático-nacional-de-defunciones-sinadef-ministerio.](https://www.datosabiertos.gob.pe/dataset/informaci%C3%B3n-defallecidos-del-sistema-inform%C3%A1tico-nacional-de-defunciones-sinadef-ministerio.)

3. [Bardach AE, Caporale JE, Alcaraz A, Augustovski F, Huayanay-Falconí L, Loza-Munarriz C, et al. Carga de enfermedad por tabaquismo e impacto potencial del incremento de precios de cigarrillos en el Perú. Rev Peru Med Exp Salud Publica [Internet]. 2016;33(4):651–61. doi:](http://paperpile.com/b/uKBKkR/kIeS5)[10.17843/rpmesp.2016.334.2548](http://dx.doi.org/10.17843/rpmesp.2016.334.2548)

4. [INEI. Instituto Nacional de Estadística e Informática [Internet]. Encuesta Nacional de Hogares (ENAHO) 2020. 2020 [citado el 18 de julio de 2022]. Disponible en:](http://paperpile.com/b/uKBKkR/mHklT) <https://datosabiertos.gob.pe/dataset/encuesta-nacional-de-hogares-enaho-2020-instituto-nacional-de-estad%C3%ADstica-e-inform%C3%A1tica-inei>

5. [World Development Indicators [Internet]. World Development Indicators. [citado 2024]. Disponible en:](http://paperpile.com/b/uKBKkR/PwXwt) <https://databank.worldbank.org/source/world-development-indicators.>

6. [Arenas de Mesa A. Los sistemas de pensiones en la encrucijada: desafíos para la sostenibilidad en América Latina y el Caribe [Internet]. Economic Commission for Latin America and the Caribbean, editor. Economic Commission for Latin America and the Caribbean; 2019 [citado el 22 de julio de 2022]. Disponible en:](http://paperpile.com/b/uKBKkR/guLlB) <https://www.cepal.org/es/publicaciones/44851-sistemas-pensiones-la-encrucijada-desafios-la-sostenibilidad-america-latina>

7. [Guindon GE, Paraje GR, Chaloupka FJ. The impact of prices and taxes on the use of tobacco products in Latin America and the Caribbean. Am J Public Health [Internet]. 2015 [citado el 22 de julio de 2024];105(3):e9–19. doi:](http://paperpile.com/b/uKBKkR/nBZjO)[10.2105/AJPH.2014.302396](http://dx.doi.org/10.2105/AJPH.2014.302396)

8. [WHO. WHO report on the global tobacco epidemic, 2019 (Tabla 9.1) [Internet]. WHO. World Health Organization; 2021 [citado el 22 de julio de 2024]. Disponible en:](http://paperpile.com/b/uKBKkR/ku1cR) <https://www.who.int/publications/i/item/WHO-HEP-HPR-TFI-2021.8.1>

9. [Wijeysundera HC, Farshchi-Zarabi S, Witteman W, Bennell MC. Conversion of the Seattle Angina Questionnaire into EQ-5D utilities for ischemic heart disease: a systematic review and catalog of the literature. Clinicoecon Outcomes Res [Internet]. 2014 [citado el 10 de septiembre de 2024];6:253–68. doi:](http://paperpile.com/b/uKBKkR/i5J3V)[10.2147/CEOR.S63187](http://dx.doi.org/10.2147/CEOR.S63187)

10. [Smith DW, Davies EW, Wissinger E, Huelin R, Matza LS, Chung K. A systematic literature review of cardiovascular event utilities. Expert Rev Pharmacoecon Outcomes Res [Internet]. 2013 [citado el 10 de septiembre de 2024];13(6):767–90. doi:](http://paperpile.com/b/uKBKkR/yqsQD)[10.1586/14737167.2013.841545](http://dx.doi.org/10.1586/14737167.2013.841545)

11. [Rutten-van Mölken M, Lee TA. Economic modeling in chronic obstructive pulmonary disease. Proc Am Thorac Soc [Internet]. 2006 [citado el 10 de septiembre de 2024];3(7):630–4. doi:](http://paperpile.com/b/uKBKkR/SDp4W)[10.1513/pats.200603-095SS](http://dx.doi.org/10.1513/pats.200603-095SS)

12. [Yeoh YS, Koh GC-H, Tan CS, Tu TM, Singh R, Chang HM, et al. Health-related quality of life loss associated with first-time stroke. PLoS One [Internet]. 2019 [citado el 10 de septiembre de 2024];14(1):e0211493. doi:](http://paperpile.com/b/uKBKkR/SZKHJ)[10.1371/journal.pone.0211493](http://dx.doi.org/10.1371/journal.pone.0211493)

13. [Pepper PV, Owens DK. Cost-effectiveness of the pneumococcal vaccine in healthy younger adults. Med Decis Making [Internet]. 2002 [citado el 10 de septiembre de 2024];22(5 Suppl):S45–57. doi:](http://paperpile.com/b/uKBKkR/xbtwc)[10.1177/027298902237705](http://dx.doi.org/10.1177/027298902237705)

14. [Chouaid C, Agulnik J, Goker E, Herder GJM, Lester JF, Vansteenkiste J, et al. Health-related quality of life and utility in patients with advanced non-small-cell lung cancer: a prospective cross-sectional patient survey in a real-world setting. J Thorac Oncol [Internet]. 2013 [citado el 10 de septiembre de 2024];8(8):997–1003. doi:](http://paperpile.com/b/uKBKkR/9r3Wb)[10.1097/JTO.0b013e318299243b](http://dx.doi.org/10.1097/JTO.0b013e318299243b)

15. [Nie M, Liu C, Pan Y-C, Jiang C-X, Li B-R, Yu X-J, et al. Development and evaluation of oral Cancer quality-of-life questionnaire (QOL-OC). BMC Cancer [Internet]. 2018 [citado el 10 de septiembre de 2024];18(1):523. doi:](http://paperpile.com/b/uKBKkR/31h01)[10.1186/s12885-018-4378-6](http://dx.doi.org/10.1186/s12885-018-4378-6)

16. [Graham AJ, Shrive FM, Ghali WA, Manns BJ, Grondin SC, Finley RJ, et al. Defining the optimal treatment of locally advanced esophageal cancer: a systematic review and decision analysis. Ann Thorac Surg [Internet]. 2007 [citado el 10 de septiembre de 2024];83(4):1257–64. doi:](http://paperpile.com/b/uKBKkR/Q1eS1)[10.1016/j.athoracsur.2006.11.061](http://dx.doi.org/10.1016/j.athoracsur.2006.11.061)

17. [Dan YY, So JBY, Yeoh KG. Endoscopic screening for gastric cancer. Clin Gastroenterol Hepatol [Internet]. 2006 [citado el 10 de septiembre de 2024];4(6):709–16. doi:](http://paperpile.com/b/uKBKkR/ZwxEy)[10.1016/j.cgh.2006.03.025](http://dx.doi.org/10.1016/j.cgh.2006.03.025)

18. [Gordois A, Scuffham P, Warren E, Ward S. Cost-utility analysis of imatinib mesilate for the treatment of advanced stage chronic myeloid leukaemia. Br J Cancer [Internet]. 2003 [citado el 10 de septiembre de 2024];89(4):634–40. doi:](http://paperpile.com/b/uKBKkR/r2X2D)[10.1038/sj.bjc.6601151](http://dx.doi.org/10.1038/sj.bjc.6601151)

19. [Pickard AS, Jiang R, Lin H-W, Rosenbloom S, Cella D. Using patient-reported outcomes to compare relative burden of cancer: EQ-5D and Functional Assessment of Cancer Therapy-General in eleven types of cancer. Clin Ther [Internet]. 2016 [citado el 10 de septiembre de 2024];38(4):769–77. doi:](http://paperpile.com/b/uKBKkR/txMMW)[10.1016/j.clinthera.2016.03.009](http://dx.doi.org/10.1016/j.clinthera.2016.03.009)

20. [Leunis A, Redekop WK, Uyl-de Groot CA, Löwenberg B. Impaired health-related quality of life in acute myeloid leukemia survivors: a single-center study. Eur J Haematol [Internet]. 2014 [citado el 10 de septiembre de 2024];93(3):198–206. doi:](http://paperpile.com/b/uKBKkR/Eykuy)[10.1111/ejh.12324](http://dx.doi.org/10.1111/ejh.12324)

21. [Hevér NV, Péntek M, Balló A, Gulácsi L, Baji P, Brodszky V, et al. Health related quality of life in patients with bladder cancer: a cross-sectional survey and validation study of the Hungarian version of the Bladder Cancer Index. Pathol Oncol Res [Internet]. 2015 [citado el 10 de septiembre de 2024];21(3):619–27. doi:](http://paperpile.com/b/uKBKkR/yB29G)[10.1007/s12253-014-9866-7](http://dx.doi.org/10.1007/s12253-014-9866-7)

22. [Endarti D, Riewpaiboon A, Thavorncharoensap M, Praditsitthikorn N, Hutubessy R, Kristina SA. Evaluation of health-related quality of life among patients with cervical cancer in Indonesia. Asian Pac J Cancer Prev [Internet]. 2015 [citado el 10 de septiembre de 2024];16(8):3345–50. doi:](http://paperpile.com/b/uKBKkR/TkVW6)[10.7314/apjcp.2015.16.8.3345](http://dx.doi.org/10.7314/apjcp.2015.16.8.3345)

23. [Sung H, Ferlay J, Siegel RL, Laversanne M, Soerjomataram I, Jemal A, et al. Global cancer statistics 2020: GLOBOCAN estimates of incidence and mortality worldwide for 36 cancers in 185 countries. CA Cancer J Clin [Internet]. 2021 [citado el 6 de mayo de 2025];71(3):209–49. doi:](http://paperpile.com/b/uKBKkR/OsRnB)[10.3322/caac.21660](http://dx.doi.org/10.3322/caac.21660)

24. [Jørgensen T, Johansen C, Jensen G, Møller H, Møller S, Pedersen N, et al. Det danske MONICA-projekt. Resultater, erfaringer og perspektiver. Ugeskr Laeger. 1999;161(44):6089–95.](http://paperpile.com/b/uKBKkR/uDZdl)

25. [Thorvaldsen P, Kuulasmaa K, Rajakangas AM, Rastenyte D, Sarti C, Wilhelmsen L. Stroke trends in the WHO MONICA project. Stroke [Internet]. 1997 [citado el 6 de mayo de 2025];28(3):500–6. doi:](http://paperpile.com/b/uKBKkR/ki4xR)[10.1161/01.str.28.3.500](http://dx.doi.org/10.1161/01.str.28.3.500)

26. [Caccavo A, Álvarez A, Bello FH, Ferrari AE, Carrique AM, Lasdica S, et al. Incidencia poblacional del infarto con elevación del ST o bloqueo de rama izquierda a lo largo de 11 años en una comunidad de la provincia de Buenos Aires. Rev Argent Cardiol [Internet]. 2007 [citado el 6 de mayo de 2025];75(3):185–8. Disponible en:](http://paperpile.com/b/uKBKkR/Wq0wS) <https://www.scielo.org.ar/scielo.php?script=sci_abstract&pid=S1850-37482007000300006&lng=es&nrm=iso&tlng=pt>

27. [Menezes AMB, Perez-Padilla R, Jardim JRB, Muiño A, Lopez MV, Valdivia G, et al. Chronic obstructive pulmonary disease in five Latin American cities (the PLATINO study): a prevalence study. Lancet [Internet]. 2005 [citado el 6 de mayo de 2025];366(9500):1875–81. doi:](http://paperpile.com/b/uKBKkR/29ojm)[10.1016/S0140-6736(05)67632-5](http://dx.doi.org/10.1016/S0140-6736(05)67632-5)

28. [Tuomilehto J, Bonita R, Stewart AW, Nissinen A, Salonen JT. Disparities in stroke mortality trends between eastern and western European MONICA populations. Stroke. 1991;22(12):1518–25.](http://paperpile.com/b/uKBKkR/rAMaf)

29. [Malyutina S, Bobak M, Kurilovitch S, Gafarov V, Simonova G, Nikitin Y, et al. Relation between heavy and binge drinking and all-cause and cardiovascular mortality in Novosibirsk, Russia: a prospective cohort study. Lancet [Internet]. 2002 [citado el 6 de mayo de 2025];360(9344):1448–54. doi:](http://paperpile.com/b/uKBKkR/MYXeO)[10.1016/S0140-6736(02)11470-X](http://dx.doi.org/10.1016/S0140-6736(02)11470-X)

30. [Rastenyte D, Tuomilehto J, Sarti C. Increasing trends in stroke mortality in Kaunas, Lithuania, between 1986 and 1995: the MONICA stroke register. Cerebrovasc Dis. 1998;8(4):210–7.](http://paperpile.com/b/uKBKkR/Vqp3m)

31. [Doll R, Peto R, Boreham J, Sutherland I. Mortality in relation to smoking: 50 years’ observations on male British doctors. BMJ [Internet]. 2004 [citado el 6 de mayo de 2025];328(7455):1519. doi:](http://paperpile.com/b/uKBKkR/8qKUS)[10.1136/bmj.38142.554479.AE](http://dx.doi.org/10.1136/bmj.38142.554479.AE)
