# Supplementary material for: Disease burden from tobacco consumption in Peru and the projected effect of strengthening control measures: a modeling study
Source: Rev Peru Med Exp Salud Publica. 2025 Jun 23;42(2):126–37. doi: 10.17843/rpmesp.2025.422.14338 (PMC12377892; doi:10.17843/rpmesp.2025.422.14338)
Supplement: Translation

# Translation: Carga de enfermedad por consumo de tabaco en Perú y efecto proyectado del fortalecimiento de medidas para su control: un estudio de modelado

**Authors:** Ariel Bardach, Andrea Alcaraz, Jhonatan R. Mejia, Natalia Espinola, Elena Lazo, Federico Cairoli, Alfredo Palacios, Lucas Perelli, Federico Augustovski, Cesar Loza-Munarriz, Agustin Casarini, Andrés Pichon-Riviere

**Affiliations:** 1 Instituto de Efectividad Clínica y Sanitaria (IECS), Buenos Aires, Argentina; 2 Centro de Investigaciones en Epidemiología y Salud Pública (CIESP), Instituto de Efectividad Clínica y Sanitaria (IECS-CONICET), Buenos Aires, Argentina; 3 Unidad de Epidemiología Clínica, Facultad de Medicina Alberto Hurtado, Universidad Peruana Cayetano Heredia. Lima, Perú.

**Keywords:** Tabaquismo, Control del tabaco, Costo de Enfermedad, Perú

## Abstract

**Objetivos.:**

Estimar la carga económica y de enfermedad asociada al consumo de tabaco en Perú y el efecto proyectado de fortalecer medidas específicas de control del tabaco.

**Materiales y métodos.:**

Utilizamos un modelo de microsimulación de Markov para evaluar la mortalidad atribuible al tabaquismo, eventos de enfermedad, costos económicos y beneficios proyectados a diez años considerando la implementación de medidas como el empaquetado neutro, el cumplimiento total de las leyes sobre lugares libres de humo, la prohibición completa de la publicidad, promoción y patrocinio del tabaco, y aumento de los impuestos sobre los cigarrillos.

**Resultados.:**

Anualmente en Perú, alrededor de 22,350 muertes y 126,000 eventos de enfermedad son atribuibles al tabaquismo, con 19% de todas las muertes por enfermedades cardíacas, 18% de las muertes por accidente cerebrovascular y 515,768 años de vida perdidos. Además, aproximadamente el 1,28% del producto bruto interno se pierde anualmente debido al tabaquismo. Durante diez años, la implementación del empaquetado neutro podría evitar 6218 muertes, 31,700 eventos y ahorrar USD 576 millones. Cumplir completamente con los lugares libres de humo evitaría 4982 muertes, 25,400 eventos y ahorraría USD 461 millones. Prohibir la publicidad, promoción y patrocinio podría prevenir 8767 muertes, 44,700 eventos y ahorrar USD 812 millones. Aumentar el precio de los cigarrillos en un 50% podría evitar 20,400 muertes, 658,400 años de vida saludables perdidos, y un beneficio económico superior a los USD 3300 millones.

**Conclusiones.:**

La carga económica y de enfermedad del uso del tabaco en Perú es importante. Un mayor esfuerzo en el control del tabaco reduciría significativamente esta carga.

## INTRODUCCIÓN

El consumo de tabaco es responsable de más de 8 millones de muertes a nivel mundial, siendo la primera causa de años de vida ajustados por discapacidad (AVAD) en varones y la séptima en mujeres durante 2019 ^1^. Desde la implementación del Convenio Marco de la Organización Mundial de la Salud (OMS) para el Control del Tabaco (CMCT) y las medidas asociadas MPOWER, se ha logrado un progreso significativo en el control del tabaco a nivel mundial ^2^. Sin embargo, el 20,9% de la población mundial de 15 años o más sigue utilizando tabaco ^3^, lo que convierte al tabaquismo en uno de los principales factores de riesgo prevenibles de morbilidad y mortalidad a nivel global. Además, la carga económica asociada al consumo de tabaco es considerablemente alta, representando el 5,7% del gasto en salud en el mundo ^4^.

Entre las regiones de la OMS, las Américas tienen la segunda prevalencia más baja del mundo (16,3%) de uso de tabaco ajustada por edad ^5^. Sin embargo, se estima que el tabaquismo causa alrededor del 14% del total de muertes en la región ^6^. En el contexto de Latinoamérica, el impacto económico del consumo de tabaco es relevante con USD 26.9 mil millones ^7^, y va más allá de los costos directos de atención médica, incluyendo también los costos sociales (productividad laboral y el cuidado informal) ^7^. Específicamente, Perú presenta una de las prevalencias más bajas de tabaquismo en adultos, con una tasa ajustada por edad estimada en 7,1% (11,6% en varones y 2,6% en mujeres) de peruanos fumadores ^3^. Además, el consumo de tabaco entre los jóvenes es relativamente bajo, con una prevalencia ajustada por edad estimada en 7,2% de jóvenes fumadores ^8^. A pesar de la baja prevalencia de tabaquismo en Perú, un estudio previo mostró que el 31% de las muertes por enfermedades asociadas con el tabaquismo se atribuyen al tabaco en 2015 ^9^, con 22.374 muertes y una carga económica estimada en USD 2.651 millones en 2020 ^10^.

Los pilares del control del tabaco en Perú son las Leyes 28705 (2006) ^11^ y 29517 (2010) ^12^, aprobadas tras la ratificación en 2004 del CMCT de la OMS ^2^, considerado legalmente en el país como un tratado de derechos humanos. Estas leyes establecieron medidas relacionadas con el control de tabaco basándose en la protección contra la exposición al humo de tabaco, la prohibición de fumar en espacios cerrados, y reguló su publicidad y patrocinio. De esta manera, el Perú ha implementado una prohibición de fumar en varios lugares cerrados y ciertas áreas privadas, así como en algunos espacios públicos al aire libre, logrando un cumplimiento sustancial con esta regulación (obteniendo una puntuación de 8 sobre 10 según la OMS) ^13^. Sin embargo, no existe una prohibición explícita para todas las áreas públicas al aire libre. En lo que respecta al empaquetado y etiquetado, se han añadido advertencias amplias (50% de la parte delantera y trasera) con todas las características apropiadas (por ejemplo, rotación, inclusión de imágenes) a los paquetes de cigarrillos. Además, aunque se han aumentado los impuestos a los cigarrillos, actualmente representan sólo alrededor del 73,3% del precio de venta al por menor ^13^, por debajo de la recomendación de la OMS del 75%, y los cigarrillos no son menos asequibles que hace una década. Por otro lado, la medida menos aplicada es la prohibición de la publicidad, promoción y patrocinio, ya que no existe una prohibición integral y aún se permiten formas de publicidad directa e indirecta (e.g., en revistas para personas mayores de 18 años). La implementación de estas medidas ha contribuido en reducir la prevalencia de tabaquismo, tal es el caso de una reducción de tabaquismo activo mayor al 6% en varones y 12% en mujeres adolescentes del 2007 al 2019 ^14^. No obstante, un estudio reportó una escasa reducción de la prematuridad luego de la implementación de las leyes para el control del tabaco, sugiriendo que estas medidas sean reforzadas ^15^.

Por lo tanto, a pesar del progreso en el control del tabaco en Perú, la carga de enfermedad y económica del tabaquismo en el país aún puede ser alta, como evidenció nuestro modelo previo con datos hasta el 2015 ^9^. Además, aún existe un margen para mejorar y fortalecer las medidas de control del tabaco (e.g., empaquetado neutro, aumento de impuestos), lo que podría reducir los costos nacionales y la carga de enfermedad. Al respecto, nuestro modelo previo solo evaluó el impacto del incremento de precios mediante impuestos, sin considerar otras medidas de control del tabaco ^9^. Por estos motivos, el objetivo de este estudio fue estimar la carga económica y de enfermedad atribuible al tabaquismo en Perú extendiendo el modelo previo hasta el 2020, así como las posibles reducciones en estas cargas que podrían lograrse con la implementación y fortalecimiento de las principales medidas de control del tabaco, tales como el empaquetado neutro con más del 80% de la superficie con advertencias de salud, el cumplimiento total de las leyes de espacios libres de humo y la prohibición completa de la publicidad, promoción y patrocinio del tabaco (PPPT).

**MENSAJE CLAVE**

Motivación para realizar el estudio. A pesar del progreso en el control del tabaco, la carga económica y de enfermedad en Perú sigue siendo alta. Fortalecer las regulaciones de espacios libres de humo, implementar empaquetado neutro, prohibir la promoción y patrocinio del tabaco, e incrementar los impuestos podrían reducirla.

Principales hallazgos. El tabaquismo causa 22,350 muertes y 126.000 eventos de enfermedad anualmente en Perú, resultando en una pérdida del 1,28% del PBI. Fortalecer las políticas antitabaco evitarían miles de muertes y ahorrarían miles de millones en costos.

Implicaciones. Políticas más estrictas de control del tabaco pueden reducir los costos de salud y económicos asociados al tabaquismo para el avance de la salud pública y la sostenibilidad económica en Perú.

## MATERIALES Y MÉTODOS

### Modelo económico

Estudio de modelado utilizando un modelo de Markov de enfermedad y económico de primer orden desarrollado en el Instituto de Efectividad Clínica y Sanitaria (IECS, Buenos Aires, Argentina) y programado en Microsoft Excel (Microsoft, Redmond, WA). Se realizó este modelo debido a que incluye características específicas de los individuos considerando la variabilidad individual y las interacciones entre los pacientes, las intervenciones y los eventos clínicos, representando mejor eventos históricos y diferentes en el tiempo teniendo en cuenta eventos recurrentes, así como poder incluir los costos y las utilidades respectivas ^16^^,^^17^. Este modelo ya ha sido utilizado para estimar la carga de enfermedad y económica, así como el impacto de la implementación de diferentes políticas de control de tabaco en otros países de Latinoamérica ^7^^,^^18^^-^^20^ y de otras regiones ^21^. El modelo principal compara los resultados (es decir, de enfermedad y económicos) en una cohorte simulada de peruanos que nunca han fumado con los estimados por el modelo para Perú, de acuerdo con los parámetros demográficos, epidemiológicos y financieros nacionales (Tabla Suplementaria 1).

Se puede encontrar más información sobre el desarrollo y la validación del modelo en publicaciones previas ^9^^,^^10^^,^^18^^,^^22^. En este artículo, extendemos el modelo hasta el 2020 y consideramos tres escenarios: el empaquetado neutro con más del 80% de la superficie con advertencias de salud, el cumplimiento total de las leyes de espacios libres de humo y la prohibición completa de la PPPT.

### Datos epidemiológicos

Los datos se obtuvieron a partir de una extensa búsqueda bibliográfica en MEDLINE, EMBASE, Cochrane Central, SocINDEX, EconLit, LILACS, NBER, CRD, el equipo de revisión de tabaco de Cochrane y literatura gris para encontrar datos del Ministerio de Salud del Perú, datos de la OMS y ponencias en congresos. Para ello, se utilizaron términos libres y controlados en inglés y en español relacionados al tabaquismo (“tobacco”, “cigarette”, “smoking”), a la prevalencia (“prevalence”, “burden”) de cada evento de enfermedad, y a la mortalidad (“mortality”) de las causas de muerte estudiadas (“myocardial infarction”, “stroke”, “pneumonia”, “COPD”, “chronic obstructive pulmonary disease”, “lung cancer”, ”oropharynx cancer”, “esophageal cancer”, “gastric cancer”, “pancreatic cancer”, “kidney cancer”, “laryngeal cancer”, “cervix cancer”, “bladder cancer”, y “leukemia”). Estos términos fueron unidos con operadores *booleanos* y traducidos a cada base de datos.

Se estimaron el número de casos y porcentajes de mortalidad anual atribuible al tabaquismo, tanto en general como estratificados por causa según sus CIE 10 correspondientes (es decir, infarto agudo de miocardio [IAM] [CIE 10: I210-I229]; resultados coronarios no IAM [CIE 10: I200-I209]; accidente cerebrovascular [CIE 10: I60, I61, I63, I64, I620, I621, I629, I678, I679, I690-I694, I698]; neumonía [CIE 10: J100-J189]; enfermedad pulmonar obstructiva crónica [EPOC] [CIE 10: J400-J439, J44X]; y cáncer de pulmón [CIE 10: C330-C349], orofaríngeo [CIE 10: C000-C009, C140, C142, C148], esofágico [CIE 10: C150-C159], estomacal [CIE 10: C160-C169], pancreático [CIE 10: C250-C259], renal [CIE 10: C64X-C65X], laríngeo [CIE 10: C320-C329], cuello uterino [CIE 10: C530-C539], de vejiga [CIE 10: C670-C679], y leucemia [CIE 10: C920]), número de casos y porcentajes de eventos de enfermedad atribuibles a patologías específicas causadas por el tabaquismo, y AVAD (es decir, la suma de años de vida perdidos [AVP] y años vividos con discapacidad [AVD]) mediante la simulación de la duración total de la vida de cada individuo para obtener los resultados totales. Todos ellos fueron estratificados por sexo (es decir, varones y mujeres).

El modelo no estimó la carga de los efectos perinatales ni de la exposición al humo ambiental. Sin embargo, se asumió que estos factores contribuyen con una carga adicional del 12% para las mujeres y del 13,6% para los varones ^23^. Las incidencias se obtuvieron para eventos de enfermedad agudos, mientras que las probabilidades de desarrollar enfermedades crónicas se estimaron con una aproximación de las tasas anuales de mortalidad y supervivencia a partir de registros nacionales. Además, se evaluó un riesgo individual para cada evento o muerte.

### Calibración y validación

El proceso de calibración y validación se llevó a cabo comparando la mortalidad específica por enfermedades atribuibles al tabaco, según sexo y edad, predicha por el modelo con las estadísticas nacionales de Perú ^24^. La información de mortalidad se obtuvo del Sistema Nacional de Defunciones (SINADEF) ^24^ explorando las causas y la desagregación por edad y sexo. Las predicciones de mortalidad fueron aceptadas si se encontraban dentro de una desviación del 15% con respecto a los datos de referencia; las desviaciones que superaban este umbral llevaron a ajustes en las ecuaciones de riesgo. Además, los resultados del modelo fueron validados externamente utilizando estudios epidemiológicos y clínicos independientes que no estuvieron involucrados en el desarrollo inicial de las ecuaciones ^7^^,^^22^^,^^25^^-^^33^. En el Material Suplementario se presentan más detalles sobre la calibración y validación del modelo (Figura S1 y S2).

### Parámetros económicos

Costos médicos directos

Los costos médicos directos (consultas, diagnóstico, hospitalizaciones, y tratamiento) se extrajeron de una investigación previa realizada por nuestro equipo ^9^ y se actualizaron por inflación (tasa de inflación peruana entre 2015 y 2020 = 10,47%) para expresar todos los valores en moneda local de 2020 y luego se transformaron a USD (tipo de cambio enero 2020 = PEN 3,3) ^34^ para el manuscrito actual. Se empleó una metodología mixta de costos basada en protocolos nacionales, producto bruto interno (PBI) *per cápita* y un método Delphi. Los datos provinieron de hospitales públicos y clínicas privadas, ajustándose por inflación a valores de 2020. Más detalles metodológicos respecto al cálculo de los costos médicos directos se pueden encontrar en nuestra publicación previa ^9^^,^^18^.

Costos por pérdida de productividad laboral

El cálculo de los costos por pérdida de productividad laboral se basó en el enfoque de capital humano, que considera dos factores principales: (i) la muerte prematura de los individuos y (ii) la disminución de la productividad en el trabajo debido a eventos de salud (presentismo). Para estimar el costo de la muerte prematura, calculamos la pérdida de productividad laboral de un individuo como el valor presente de sus ingresos laborales futuros, utilizando la fórmula del Valor de una Vida Estadística ^35^. Esta fórmula permite estimar las pérdidas de productividad a través de los ingresos laborales que la sociedad pierde debido a la muerte prematura de los trabajadores. En cuanto a los costos de presentismo, el mismo se aproxima como costo debido a discapacidad, la cual se mide a través de las pérdidas de calidad de vida por cada evento en salud de acuerdo a los parámetros mostrados en la Tabla S2. Los salarios de mercado anuales por sexo y edad se estimaron utilizando una ecuación de Mincer ^36^^,^^37^ con datos nacionales representativos de la Encuesta Nacional de Hogares (ENAHO) 2020 ^38^. Para ello, se utilizaron las variables de ingreso laboral o salario, años de educación, edad, y sexo de esta encuesta nacional. Luego aplicamos la tasa de crecimiento salarial esperada del Banco Mundial, una tasa de descuento del 5% y las edades oficiales de jubilación en Perú por sexo. Para más detalles, consulte la Tabla S1. Utilizamos un método de estimación indirecta basado en investigaciones previas ^39^^,^^40^ para estimar las pérdidas económicas debido al presentismo. Específicamente, asumimos que la reducción en la productividad laboral de los individuos es directamente proporcional a la disminución de su calidad de vida debido a las condiciones de salud atribuidas al tabaquismo ^41^. Para más información y una aplicación de esta metodología, consulte Pinto *et al.*^20^ y la Tabla S2.

Costo de cuidado informal

El cuidado informal incluye horas no remuneradas proporcionadas por familiares o amigos, principalmente mujeres. Debido a la falta de microdatos específicos dedicados al cuidado en Perú sobre tiempo, este estudio empleó la metodología desarrollada por Espinola *et al*. ^42^ para estimar el tiempo de cuidado informal para pacientes con enfermedades atribuibles al tabaquismo. Para el valor del tiempo requerido para el cuidado informal, los datos salariales por hora para trabajadores de asistencia social y de salud ^38^ sirven como proxy para el costo de oportunidad. Para más detalles, consulte Espinola *et al*^42^.

Beneficios en salud y económicos proyectados

Se estudió el beneficio acumulado proyectado a 10 años sobre la carga de enfermedad y económica de la implementación de: 1) empaquetado neutro y más del 80% de la superficie de los paquetes con advertencias de salud basadas en imágenes; 2) prohibición total de la PPPT; 3) cumplimiento total de las leyes sobre lugares libres de humo; 4) y aumento del 25%, 50% y 75% en el precio minorista de los cigarrillos a través de impuestos.

Para estimar el impacto de las medidas de control del tabaco, utilizamos la metodología detallada en nuestros estudios previos ^10^. El efecto se estimó sobre la prevalencia del tabaquismo basado en la siguiente fórmula:

*Prev*_*p*_ *ost =Prev*_*p*_ *re - (Em* * *I*_*p*_ * *Prev*_*p*_ *re)*

Donde *Prev*_*p*_ *re* es la prevalencia de fumadores antes de la intervención, *Em* es la efectividad de la intervención expresada como reducción relativa en el consumo de tabaco, y *I*_*p*_ es la proporción de variación en el consumo que impacta en la prevalencia de fumadores. Diferentes estudios han estimado que, a corto y mediano plazo, aproximadamente la mitad de la reducción en el consumo es consecuencia de la reducción en la prevalencia y la otra mitad se explica por la reducción del consumo en los fumadores continuos ^43^^-^^45^. En caso de impuestos, *Em* representa el efecto del cambio de precio sobre la medida de consumo a través de la elasticidad-precio de la demanda. La efectividad de las intervenciones libres de humo también consideró la reducción del riesgo para los no fumadores debido a la disminución de la exposición al tabaquismo pasivo. Los impactos económicos y de salud se estimaron comparando los resultados predichos por el modelo para Perú con las tasas actuales de tabaquismo con los de reducción del consumo después de la intervención. Además, no se consideraron cambios en la población, ni en los costos de tratamiento ni en los salarios utilizados para la productividad perdida. Por lo tanto, los ahorros estimados corresponden a un estado estacionario donde solo ocurren cambios en la prevalencia debido a la política. El impacto de las intervenciones se informa como un efecto acumulativo a lo largo de 10 años. 

### Ética

Este estudio no requirió aprobación ética porque utilizó bases de datos públicas y artículos publicados durante el desarrollo del modelo.

## RESULTADOS

Estimamos alrededor de 22,350 muertes atribuibles al consumo de tabaco en Perú, aproximadamente el 22,4% de la mortalidad anual total del país y el 37,1% de las muertes totales por patologías asociadas al tabaquismo en personas mayores de 35 años. Aproximadamente, el 19% de todas las muertes por enfermedades cardíacas y el 18% de las muertes por accidente cerebrovascular se pueden atribuir al tabaquismo. Los porcentajes atribuibles específicos de cada enfermedad son más altos en el cáncer de laringe (84,6%), cáncer de pulmón (83,2%) y EPOC (80,1%), incluso cuando se estratifica por sexo. Además, el 21% de las muertes por neumonía se pueden atribuir al tabaquismo, y 2574 muertes se atribuyen al tabaquismo pasivo (Tabla 1).

**Tabla 1:** Carga anual de enfermedad atribuible al consumo de tabaco en Perú, 2020.

Condición	Muertes totales	Muertes atribuibles al consumo de tabaco						Eventos de enfermedad totales	Eventos de enfermedad atribuibles al consumo de tabaco					
		Total		Varones		Mujeres			Total		Varones		Mujeres	
		n	%	n	%	n	%		n	%	n	%	n	%
Infarto agudo de miocardio	6348	1303	20,5	841	23,4	462	16,8	20716	5063	24,4	3700	27,6	1363	18,7
Enfermedad cardíaca isquémica	794	189	23,9	121	25,8	68	21,1	12902	3575	27,7	2280	29,2	1295	25,4
Enfermedad cardíaca no isquémica*	4599	759	16,5	530	22	228	10	-	-	-	-	-	-	-
Accidente cerebrovascular	8288	1538	18,6	912	22,5	626	14,8	53016	10655	20,1	6006	23,6	4649	16,9
Cáncer de pulmón	2910	2420	83,2	1324	89,7	1096	76,5	3294	2730	82,9	1455	89,6	1275	76,4
Neumonía	13719	2874	21	1684	24,2	1190	17,6	110662	24169	21,8	13385	24,6	10784	19,2
EPOC	9517	7625	80,1	3910	81,5	3715	78,7	103633	74959	72,3	39419	74,2	35540	70,4
Cáncer orofaríngeo	565	368	65,1	231	78,1	137	50,8	1194	777	65,1	472	77,1	305	52,4
Cáncer esofágico	366	260	71,3	186	73,5	74	66,3	461	325	70,6	232	73,2	93	64,8
Cáncer gástrico	4692	1041	22,2	749	29,5	292	13,6	5877	1314	22,4	951	29,6	363	13,6
Cáncer pancreático	1561	457	29,3	242	30,2	215	28,4	1808	530	29,3	281	30,2	249	28,4
Cáncer renal	808	237	29,4	221	41,5	16	6	1518	452	29,7	416	42,1	36	6,8
Cáncer laríngeo	136	115	84,6	86	85,7	29	81,1	286	234	81,8	177	84,2	57	75
Leucemia	1204	209	17,4	153	25	56	9,4	1742	308	17,7	224	25,3	84	9,8
Cáncer vesical	384	165	43	122	49,4	43	31,3	910	397	43,6	302	49,2	95	32,1
Cáncer de cérvix	1811	236	13	NA	NA	236	13	3819	508	13,3	NA	NA	508	13,3
Exposición al humo ambiental y otras causas	2574	2557	100	1539	100	1018	100	NA	NA	NA	NA	NA	NA	NA
Total	60275	22353	37,1	12581	41,9	9502	32,1	321840	125996	39,1	69300	-	56696	-

n: frecuencia absoluta; EPOC: enfermedad pulmonar obstructiva crónica; NA: no aplicable.

*No se presentan eventos de enfermedad atribuibles al tabaco para las enfermedades cardíacas no isquémicas debido a la falta de medidas relativas que permitan su cálculo en las fuentes de datos consultadas.

Nota: El porcentaje de muertes específicas por condición son la proporción de muertes atribuibles al consumo de tabaco del total de muertes específicas por condición en la población de 35 años o más (e.g. muertes causadas por infarto agudo de miocardio en varones: 841/23.4% significa que ocurren 841 muertes por infarto agudo de miocardio atribuibles al tabaquismo en varones, lo que representa el 23.4% del total de muertes causadas por esa condición en varones). El porcentaje de eventos de enfermedad específicos por condición son la proporción de eventos atribuibles al consumo de tabaco del total de eventos específicos por condición (e.g. eventos causados por infarto agudo de miocardio en varones: 3700/27.6% significa que ocurren 3700 eventos de infarto agudo de miocardio en varones atribuibles al tabaquismo, lo que representa el 27.6% del total de eventos de esa condición en varones). Los resultados fueron obtenidos del modelo elaborado que usó los parámetros mostrados en la Tabla S1 del material suplementario, y que fueron obtenidos tras la búsqueda bibliográfica mencionada en la subsección de Datos epidemiológicos.

Además, estimamos un total de 126,000 eventos de enfermedad anuales atribuibles al tabaquismo (39,1% del total de patologías asociadas al tabaquismo). Entre varones y mujeres, los eventos de enfermedad más frecuentes atribuidos al tabaquismo fueron cáncer de pulmón (89,6% y 76,4%), cáncer de laringe (84,2% y 75%), cáncer orofaríngeo (77,1% y 52,6%) y EPOC (74,2% y 70,4%) (Tabla 1).

### Esperanza de vida y calidad de vida asociadas al tabaquismo

La esperanza de vida para los varones fumadores fue 6,5 años más corta que para los no fumadores, y para los ex fumadores masculinos fue 3,2 años más corta. Entre las mujeres, las fumadoras tenían una esperanza de vida 7,5 años más corta que las no fumadoras, y las ex fumadoras experimentaron una reducción de 3,1 años. De esta manera, se estimó que 715,158 AVAD, con 515,768 AVP y 199,801 AVD (107.948 para varones y 91,853 para mujeres) son atribuibles al tabaquismo anualmente en Perú.

### Costos asociados al tabaquismo

La carga económica estimada del tabaquismo en Perú es de aproximadamente USD 2804 millones, con USD 1285 millones atribuidos a costos médicos directos (45,8%), USD 453 millones a pérdidas de productividad por discapacidad (16,2%), USD 325 millones a muerte prematura (11,6%) y USD 741 millones a costos de cuidados informales (26,4%). La EPOC representó el costo más importante (USD 1194 millones), seguida por el accidente cerebrovascular (USD 391 millones) y otros tipos de cáncer (USD 365 millones). La carga económica estimada representó el 1,28% del producto interno bruto, y el costo directo atribuido al tabaquismo representó el 0,59% del PBI (Tabla 2 y Figura 1).

**Tabla 2:** Carga económica anual atribuible al consumo de tabaco en Perú según sexo, condición de salud y tipo de costos, 2020.

Tipo de costo		Sexo	Costos atribuibles (millones de USD^a^)							
			EPOC	Enfermedades cardiovasculares	Cáncer de pulmón	Otros tipos de cáncer	Tabaquismo pasivo y otras causas	ACV	Neumonía	Total
Costos médicos directos		Varones	231,0	48,3	71,1	73,7	60,3	75,9	2,1	562,4
		Mujeres	247,1	107,0	70,6	122,0	86,5	86,9	2,6	722,7
		Total	478,0	155,3	141,7	195,8	146,8	162,9	4,8	1285,2
Costos de pérdida de productividad laboral	Mortalidad prematura	Varones	46,3	30,9	24,3	45,9	25,1	23,4	13,6	209,5
		Mujeres	32,5	8,1	18,1	25,0	12,4	13,8	5,7	115,6
		Total	78,8	39,1	42,4	70,8	37,5	37,3	19,3	325,1
	Discapacidad	Varones	144,6	20,2	10,7	27,9	32,9	38,1	0,1	274,5
		Mujeres	101,8	5,2	8,5	13,7	19,1	30,2	0,0	178,5
		Total	246,3	25,4	19,2	41,6	52,0	68,3	0,1	453,0
Costos del cuidado informal		Varones	201,4	36,8	12,3	35,7	48,2	64,0	4,4	402,9
		Mujeres	189,2	16,7	12,2	21,0	36,2	58,6	3,6	337,6
		Total	390,6	53,6	24,5	56,8	84,4	122,6	8,0	740,5
Total			1193,8	273,3	227,7	365,0	320,6	391,1	32,2	2803,7

EPOC: Enfermedad pulmonar obstructiva crónica; ACV: Accidente cerebrovascular.

a Los valores monetarios se expresan en USD del 2020. Tasa de cambio enero 2020: USD 1 = PEN 3,3 (Soles)

**Figura 1:** Carga económica anual atribuible al consumo de tabaco en Perú según condición y tipo de gasto (millones de USDa), 2020

‡EPOC: Enfermedad pulmonar obstructiva crónica; §Enfermedades cardiovasculares: Infarto agudo de miocardio, cardiopatía isquémica, infarto no miocárdico, enfer-medad cardiovascular no isquémica. ¶Otros tipos de cáncer: Oral, estómago, esófago, páncreas, riñón, vejiga, laringe, cérvix, y leucemia.a Los valores monetarios se expresan en USD del 2020. Tasa de cambio enero 2020: USD 1 = PEN 3,3 (Soles)

### Beneficios esperados del fortalecimiento de las medidas de control del tabaco

En diez años, el empaquetado neutro evitaría 6218 muertes, 31,700 eventos, 200,900 AVAD y el gasto de USD 576 millones atribuidos al tabaquismo. Además, el cumplimiento total de las leyes sobre ambientes libres de humo evitaría 4982 muertes, 25,400 eventos, 160,959 AVAD y el gasto de USD 461 millones atribuidos al tabaquismo. Asimismo, la prohibición total de la PPPT evitaría 8767 muertes, 44,700 eventos, 283,270 AVAD y y el gasto de USD 812 millones atribuidos al tabaquismo. La mayoría de las muertes y eventos evitados en los tres escenarios ocurrieron en EPOC y neumonía atribuibles al tabaquismo, mientras que la reducción de costos se observó principalmente en EPOC y accidentes cerebrovasculares (Tabla 3).

**Tabla 3:** Escenarios proyectados a diez años de reducción de mortalidad, eventos y costos asociados con el fortalecimiento de las medidas de control del tabaco, 2020-2029.

Escenarios		Reducción en mortalidad		Reducción en eventos		Reducción en costos (millones de USD^a^)	
		Caso base (LI - LS)	Reducción estimada (LI - LS)	Caso base (LI - LS)	Reducción estimada (LI - LS)	Caso base (LI - LS	Reducción estimada (LI - LS)
Empaquetado neutro y más del 80% de la superficie con advertencias sanitarias							
	Enfermedades cardiovasculares	646 (323-970)	688 (361-1742)	2525 (1263-3788)	2687 (1410-6806)	62,82 (31,41-94,24)	66,83 (35,09-169,31)
	Accidente cerebrovascular	670 (335-1005)	712 (374-1805)	4606 (2303-6909)	4900 (2573-12414)	123,38 (61,69-185,07)	131,25 (68,91-332,51)
	EPOC	1539 (769-2308)	1637 (859-4147)	13202 (6601-19803)	14044 (7373-35579)	177,39 (88,69-266,08)	188,71 (99,07-478,06)
	Neumonía	861 (430-1291)	915 (481-2319)	7461 (3731-11192)	7937 (4167-20108)	3,94 (1,97-5,91)	4,19 (2,2-10,62)
	Cáncer de pulmón	678 (339-1017)	721 (379-1827)	762 (381-1144)	811 (426-2055)	46,49 (23,25-69,74)	49,46 (25,97-125,3)
	Otros tipos de cáncer	779 (389-1168)	829 (435-2099)	1247 (624-1871)	1327 (696-3361)	65,54 (32,77-98,31)	69,72 (36,6-176,63)
	Exposición al humo ambiental y otras causas	672 (336-1009)	715 (376-1812)	NA	NA	61,83 (30,92-92,75)	65,78 (34,54-166,65)
	Total	5845 (2922-8767)	6218 (3265-15751)	29804 (14902-44706)	31706 (16645-80323)	541,4 (270,7-812,1)	575,96 (302,38-1459,09)
Cumplimiento total de las leyes antitabaco							
	Enfermedades cardiovasculares	1129 (572-1671)	551 (236-1052)	3788 (2104-9891)	2152 (922-4108)	109,76 (55,62-162,41)	53,55 (22,94-102,2)
	Accidente cerebrovascular	1170 (593-1731)	571 (245-1089)	6909 (3838-18041)	3926 (1682-7493)	215,56 (109,24-318,96)	105,16 (45,05-200,7)
	EPOC	2689 (1363-3978)	1312 (562-2503)	19803 (11001-51707)	11252 (4820-21475)	309,91 (157,05-458,57)	151,19 (64,76-288,55)
	Neumonía	1503 (762-2225)	733 (314-1400)	11192 (6218-29223)	6359 (2724-12137)	6,89 (3,49-10,19)	3,36 (1,44-6,41)
	Cáncer de pulmón	1184 (600-1752)	578 (247-1103)	1144 (635-2986)	650 (278-1240)	81,23 (41,17-120,2)	39,63 (16,98-75,63)
	Otros tipos de cáncer	1361 (690-2014)	664 (284-1267)	1871 (1039-4884)	1063 (455-2029)	114,51 (58,03-169,43)	55,86 (23,93-106,61)
	Exposición al humo ambiental y otras causas	1175 (595-1738)	573 (246-1094)	NA	NA	108,03 (54,75-159,85)	52,7 (22,58-100,58)
	Total	10212 (5175-15110)	4982 (2134-9508)	44706 (24836-116731)	25402 (10881-48482)	945,88 (479,34-1399,62)	461,45 (197,67-880,68)
Prohibición total de PPPT^b^							
	Enfermedades cardiovasculares	108 (0-1465)	970 (539-2532)	421 (0-5724)	3788 (2104-9891)	10,47 (0-142,4)	94,24 (52,35-246,06)
	Accidente cerebrovascular	112 (0-1518)	1005 (558-2623)	768 (0-10441)	6909 (3838-18041)	20,56 (0-279,66)	185,07 (102,82-483,23)
	EPOC	256 (0-3488)	2308 (1282-6027)	2200 (0-29924)	19803 (11001-51707)	29,56 (0-402,07)	266,08 (147,82-694,76)
	Neumonía	143 (0-1951)	1291 (717-3370)	1244 (0-16912)	11192 (6218-29223)	0,66 (0-8,93)	5,91 (3,28-15,44)
	Cáncer de pulmón	113 (0-1537)	1017 (565-2655)	127 (0-1728)	1144 (635-2986)	7,75 (0-105,39)	69,74 (38,75-182,1)
	Otros tipos de cáncer	130 (0-1766)	1168 (649-3051)	208 (0-2827)	1871 (1039-4884)	10,92 (0-148,56)	98,31 (54,62-256,7)
	Exposición al humo ambiental y otras causas	112 (0-1524)	1009 (560-2634)	NA	NA	10,31 (0-140,16)	92,75 (51,53-242,19)
	Total	974 (0-13248)	8767 (4871-22892)	4967 (0-67555)	44707 (24835-116732)	90,23 (0-1227,17)	812,1 (451,17-2120,48)

LI: límite inferior; LS: límite superior; EPOC: enfermedad pulmonar obstructiva crónica; NA: no aplicable; PPPT: publicidad, promoción y patrocinio del tabaco

a Los valores monetarios se expresan en USD del 2020. Tasa de cambio enero 2020: USD 1 = PEN 3,3 (Soles)

b El caso base corresponde a una prohibición amplia de PPPT y la reducción estimada corresponde a una prohibición completa de PPPT.

Adicionalmente, alrededor de 10,180, 20,400, 30,500 muertes y 329,200, 658,400 y 987,700 años de vida saludable perdidos serían evitados mediante un aumento del 25%, 50% y 75% en el precio de los cigarrillos. Los tres escenarios de aumento de precio ahorrarían USD 1717, USD 3300 y USD 4752 millones en diez años, respectivamente (Tabla 4 y Figura 2).

**Tabla 4:** Beneficios económicos y de salud acumulados durante 10 años de un aumento del 25%, 50% y 75% en el precio minorista de los cigarrillos a través de impuestos en Perú, 2020-2029.

Beneficios		Caso base Incremento de precio		
		25%	50%	75%
Efectos en salud (n)				
	Muertes evitadas	10180	20359	30539
	Años de vida saludable perdidos evitados	329221	658443	987664
	Eventos de enfermedad cardíaca coronaria evitados	4398	8797	13195
	Eventos de accidentes cerebrovasculares evitados	8022	16045	24067
	Eventos de EPOC evitados	22993	45986	68979
	Eventos de cáncer evitados	3500	7000	10499
Efectos económicos (millones de USD^a^)				
	Ahorro en costos de atención sanitaria	600	1201	1801
	Ahorro en costos por pérdida de productividad laboral	336	672	1007
	Ahorro de costos para cuidadores informales	343	685	1028
	Aumento de los ingresos fiscales	438	743	915
Beneficio económico total		1717	3300	4752

a Los valores monetarios se expresan en USD del 2020. Tasa de enero 2020: USD 1 = PEN 3,3 (Soles)

**Figura 2:** Análisis comparativo del impacto proyectado de tres escenarios diferentes de aumento en el precio de los cigarrillos, tanto en el número estimado de muertes evitadas como en el ahorro de costos económicos durante diez años en Perú (millones de USDa), 2020-2029.

n: frecuencia absoluta ^a^ Los valores monetarios se expresan en USD del 2020. Tasa de cambio enero 2020: USD 1 = PEN 3,3 (Soles)

## DISCUSIÓN

Según nuestros resultados, aproximadamente 22,350 peruanos mueren cada año debido al consumo de tabaco, lo que representa más del 22,4% del total de muertes anuales en el Perú. Además, 126,000 eventos fueron atribuibles al consumo de tabaco, con una alta carga debida a la EPOC, el cáncer de pulmón, la neumonía, una pérdida total de más de 515 mil años de vida perdidos y más de 199 mil años de vida saludable. Además, la carga económica atribuible al consumo de tabaco alcanzó los USD 2804 millones, aproximadamente el 1,28% del PBI de 2020.

En nuestro modelo, cada medida de control del tabaco contribuyó a una reducción considerable en las muertes (entre 4900 a 8700), eventos (entre 25400 a 44700) y costos atribuibles (entre USD 461 millones y USD 812 millones) al consumo de tabaco. Un escenario de aumento del 50% redujo significativamente las muertes (20,300 muertes) y aumentó el número de años de vida saludable perdidos evitados (658,000 años) en un periodo de diez años si se mantienen los beneficios actuales.

En Latinoamérica, se atribuyeron 350,593 muertes y 2,248,394 eventos por año al consumo de tabaco en 2020 entre doce países ^7^. Brasil y México encabezaron esta lista, mientras que Perú ocupó el quinto lugar ^7^. Además, en 2016, estimamos la carga de enfermedad del consumo de tabaco en Perú, con 16,719 muertes anuales y 95,665 eventos anuales atribuibles al tabaquismo, lo que representó el 12,5% de las muertes totales, menos que nuestra estimación actual ^9^. Esto podría deberse a mayores tasas de prevalencia de enfermedades atribuibles al tabaquismo, como la EPOC (un aumento del 24,6% en las tasas de prevalencia estandarizadas por edad) ^46^ y el cáncer de pulmón (un aumento absoluto del 93%)^47^ reportados desde 1990 hasta 2019, debido a una mejor y más temprana detección, los esfuerzos para incrementar la detección temprana de otras enfermedades atribuibles al tabaco y la influencia simultánea de otros factores de riesgo como la exposición a biomasa, la tuberculosis, entre otros ^48^.

La carga económica total atribuida al tabaco en los países de Latinoamérica fue de USD 49,804 millones, con Brasil a la cabeza de la lista, seguido por México, mientras que Perú ocupó el quinto lugar ^7^. Nuestra estimación de 2016 calculó una carga económica anual en Perú de PEN 2535 millones, lo que representaba el 0,4% del PBI ^9^. Sin embargo, esta cifra no incluía los costos financieros indirectos. La estimación actual muestra un aumento a USD 2803 millones, con los costos indirectos comprendiendo el 52,7% de la carga económica total, representando un mayor uso del PBI de Perú (1,28%). Las enfermedades respiratorias y los cánceres lideran la carga financiera, seguidas por los accidentes cerebrovasculares. Por otro lado, el tabaquismo pasivo y otras causas contribuyeron con USD 320 millones (11,4%), la mayoría proveniente de los costos directos y de cuidados informales. Resultados similares fueron reportados para Argentina, Brasil, Chile, Colombia y Ecuador ^7^, lo que destaca la necesidad de discutir los costos sociales atribuibles al tabaco durante las discusiones de políticas, como el impacto desproporcionado en las mujeres debido a su rol en los cuidados informales ^49^.

Aunque Perú ha tomado medidas necesarias para el control del tabaco en las últimas décadas ^11^^-^^13^, aún enfrenta desafíos para reducir su impacto económico y sanitario. Según nuestro modelo, las estrategias más efectivas incluyen una regulación más estricta de la prohibición de la PPPT, un aumento en los impuestos a los cigarrillos para reducir su accesibilidad, y una mayor adherencia a las leyes de espacios libres de humo ^13^. Además, el empaquetado neutro con más del 80% de la superficie con advertencias de salud también contribuyó a una reducción de muertes, eventos y costos atribuibles al tabaquismo. Estos hallazgos coinciden con estudios de otros países de Latinoamérica ^18^^,^^50^, en los que las medidas para el control del tabaco proyectaron una reducción considerable de la carga económica y de enfermedad. Esto evidencia que los países de Latinoamérica comparten una brecha importante de mejora que conduciría a resultados favorables si fuera abordada con medidas más estrictas ^18^^,^^50^.

Si bien, Perú ha logrado un nivel adecuado de advertencia sobre los peligros del tabaco, aún no cumple completamente con las prohibiciones de PPPT y las leyes de espacios libres de humo, obteniendo ocho de diez puntos en estas áreas ^13^. Para lograr su cumplimiento total, se requiere una prohibición explícita y completa del tabaquismo en lugares públicos al aire libre. Según la OMS, la regulación debe enfocarse en universidades, áreas exteriores, vehículos privados con niños, áreas de juegos infantiles al aire libre, bares y pubs; lugares en los que Perú alcanzó entre tres a cinco puntos de un total de diez ^13^. Para fortalecer estas medidas, se recomienda habilitar números de teléfono u otros mecanismos para denunciar infracciones, multas a los establecimientos que no retiren ceniceros, asignar fondos para la aplicación de normativas y prohibir explícitamente los productos de tabaco calentado y los dispositivos de nicotina, tanto electrónicos como no electrónicos ^13^.

Considerando que el aumento de impuestos sobre los cigarrillos es la medida más rentable para reducir el consumo de tabaco ^51^, nuestro modelo mostró que un incremento del 50% en el precio reduciría significativamente las muertes y los años de vida saludable perdidos en diez años. Nuestros resultados indican que mayores aumentos de precio generan un mayor impacto en la reducción del consumo y la mortalidad asociada, siendo el incremento del 75% el que produjo los mayores beneficios económicos, los cuales se amplifican con el tiempo. Esto sugiere que los efectos positivos de un alza en los precios no solo son inmediatos, sino que se acumulan a lo largo de los años. Aunque Perú ha logrado un progreso notable al aumentar los impuestos de los cigarrillos al 73,3%, aún no alcanza el 75% recomendado por la OMS, debiendo ajustar el impuesto según la inflación para evitar la reducción real del precio ^13^. A pesar de actualizar su impuesto al tabaco en enero de 2024 ^52^, el Perú aún no ha alcanzado esta meta.

Los resultados de nuestro estudio deben interpretarse a la luz de algunas limitaciones. El modelo no incluye el impacto de productos alternativos al tabaco, como los cigarrillos electrónicos y el tabaco calentado, lo que podría subestimar la carga real de la epidemia de nicotina en Perú. Cabe destacar que los sistemas electrónicos de suministro de nicotina o sin nicotina no estuvieron regulados en el país durante el periodo del estudio. Por otro lado, los costos de atención médica se estimaron a partir de información de expertos, guías de práctica clínica locales y literatura especializada, lo que podría no reflejar diferencias regionales en diagnóstico, tratamiento y seguimiento, afectando la precisión de la estimación de costos en el sistema de salud. Además, el modelo representa resultados a nivel nacional, es probable que existan variaciones locales o regionales.

Adicionalmente, varios costos fuera del sector sanitario no fueron incluidos, como el costo de la productividad laboral debido al ausentismo o el impacto ambiental. Además, considerar el crecimiento económico, el aumento de la productividad y los cambios demográficos podría mejorar la precisión de las proyecciones a 10 años. No obstante, los hallazgos siguen siendo valiosos, ya que pueden interpretarse como un escenario de estado estable o como el valor presente de los beneficios a lo largo de la década. Asimismo, no tomamos en cuenta los factores de equidad al aumentar los impuestos al tabaco sobre la población, lo que podría ser mejor explorado en un análisis de costo-efectividad distributivo o un análisis de costo-efectividad extendido. Por otro lado, tampoco consideramos el impacto de la pandemia de COVID-19 durante 2020 en la prevalencia del tabaquismo y el tabaquismo pasivo debido a las restricciones de movilidad ^53^, así como de otras alteraciones sanitarias y socioeconómicas ocurridas durante este periodo ^54^. Por otra parte, las medidas de control del tabaco se analizaron de forma aislada, aunque existe evidencia de que su implementación conjunta generaría efectos sinérgicos, lo que podría potenciar los beneficios estimados ^55^. Finalmente, no se realizó una revisión sistemática para la búsqueda de la información incluida en el modelo, sin embargo, la búsqueda de la literatura fue exhaustiva y abarcó diferentes bases de datos incluyendo la mejor evidencia disponible al momento de la elaboración del modelo.

Por otro lado, nuestro estudio también presenta fortalezas relevantes. Dado que los datos específicos sobre la implementación de políticas difícilmente pueden ser estudiados en estudios prospectivos, nuestro modelo representa una oportunidad significativa para simular el impacto de las medidas de control del tabaco, proporcionando a los responsables de políticas en Perú datos para el proceso de toma de decisiones. Además, este estudio representa el informe más completo y actualizado que añade los costos indirectos a la carga económica.

En conclusión, la carga de enfermedad y económica del consumo de tabaco en Perú sigue siendo considerable. Para mitigar este impacto, es crucial implementar impuestos más altos a los cigarrillos, garantizar el cumplimiento total de las regulaciones de espacios libres de humo, introducir empaques neutros e imponer una prohibición integral de la publicidad, promoción y patrocinio del tabaco. Estas medidas tienen el potencial de reducir significativamente tanto la prevalencia del tabaquismo como los costos de salud y económicos asociados. Por lo tanto, priorizar estas intervenciones políticas es esencial para avanzar en la salud pública y la sostenibilidad económica en Perú.
